# Nutraceuticals: Transformation of Conventional Foods into Health Promoters/Disease Preventers and Safety Considerations

**DOI:** 10.3390/molecules26092540

**Published:** 2021-04-27

**Authors:** Mudhi AlAli, Maream Alqubaisy, Mariam Nasser Aljaafari, Asma Obaid AlAli, Laila Baqais, Aidin Molouki, Aisha Abushelaibi, Kok-Song Lai, Swee-Hua Erin Lim

**Affiliations:** 1 Health Sciences Division, Abu Dhabi Women’s College, Higher Colleges of Technology, Abu Dhabi 41012, United Arab Emirates; H00349412@hct.ac.ae (M.A.); H00349801@hct.ac.ae (M.A.); H00349760@hct.ac.ae (M.N.A.); H00323776@hct.ac.ae (A.O.A.); H00307981@hct.ac.ae (L.B.); lkoksong@hct.ac.ae (K.-S.L.); 2 Department of Avian Disease Research and Diagnostic, Razi Vaccine and Serum Research Institute, Agricultural Research Education and Extension Organization (AREEO), Karaj 31585-854, Iran; aidinmolouki@gmail.com; 3 Dubai Colleges, Higher Colleges of Technology, Dubai 16062, United Arab Emirates; aabushelaibi@hct.ac.ae

**Keywords:** functional foods, anti-cancer, anti-inflammation, antioxidant activity, anti-lipid activity, nutraceuticals safety and toxicity

## Abstract

Nutraceuticals are essential food constituents that provide nutritional benefits as well as medicinal effects. The benefits of these foods are due to the presence of active compounds such as carotenoids, collagen hydrolysate, and dietary fibers. Nutraceuticals have been found to positively affect cardiovascular and immune system health and have a role in infection and cancer prevention. Nutraceuticals can be categorized into different classes based on their nature and mode of action. In this review, different classifications of nutraceuticals and their potential therapeutic activity, such as anti-cancer, antioxidant, anti-inflammatory and anti-lipid activity in disease will be reviewed. Moreover, the different mechanisms of action of these products, applications, and safety upon consumers including current trends and future prospect of nutraceuticals will be included.

## 1. Introduction

Since ancient times, conventional food and herbal extracts have been recognized as a fundamental part of the holistic approach to achieve complete wellness and health, especially in the ancient ayurvedic system in India, in addition to traditional Chinese, Roman, and Greek medicine [1]. The Greek physician Hippocrates adopted the philosophy of food as medicine, with his renowned quote “Let food be the medicine and medicine be the food” [2]. Throughout human history, many natural sources were utilized for their healing and strength restoring effects upon consumption, such as cinnamon, saffron, honey, garlic, ginger, pomegranate, mint, and many more [3].

Nutraceuticals are known as bioactive substances that are present in common food or botanical-based sources that can be delivered in the form of dietary supplements or functional food, supplying beneficial effects in addition to the nutritional essential components [4]. Nutraceuticals comprise a wide range of bioactive derivatives accumulated in edible sources including antioxidants, phytochemicals, fatty acids, amino acids, and probiotics. With either established previously or potential effects, nutraceuticals are well-known for their role of being involved in disease treatment and prevention, anti-aging properties, and malignancy prevention. Consuming probiotics is encouraged due to its significant role in the treatment and prevention of gastroenterological diseases [5]. Garlic, for example, has been suggested as a complementary therapy for high blood pressure and cholesterol [6].

With the presence of side effects induced by some pharmaceutical drugs and the emergence of antimicrobial resistance, nutraceutical compounds have gained attention as an alternative therapeutic and preventive approach alongside the advantages of being more affordable and available. Several studies have significantly shown the beneficial effects of nutraceutical ingredients on immune system functions. Such functions include enhancing the infection response mechanism, boosting immunomodulatory activity, and contributing to reducing the impacts of autoimmune disorders and hypersensitivity. Nutraceuticals have also been shown to exert lipid-lowering, anti-inflammatory, anti-cancer and antioxidant activity [7,8,9,10].

The immune system is a sophisticated host defense system, composed of different specialized cells and protein components acting as a unit in the defensive mechanism against diseases. The immune system is divided into two subsystems, commonly known as the innate and the adaptive immune systems. Both subsystems involve humoral and cellular responses. The innate immune system is based on unspecific defense strategies and components that are present from birth, starting with the first line defense (skin and mucous) and the second line, which comprises cellular mediated (granulocytes, macrophages, and dendritic cells), as well as humoral components including cytokines and complements. On the other hand, the adaptive immune system attains the immunological memory throughout previous exposure to several antigens. Therefore, this will induce a corresponding response regulated by a series of cell-mediated responses which consists of T lymphocytes responsible for recognizing pathogens and destroying them. In addition to B lymphocytes that produce specific antibodies corresponding to antigens or pathogens, it will also provide a neutralizing effect and protection against any harm that the body might encounter [11].

When it comes to boosting external immunity, vitamin C is the one of most popularly consumed compounds. Many studies have suggested the beneficial effects of vitamin C in improving the immune system by supporting the innate and adaptive systems, augmenting defense mechanisms such as phagocytosis and chemotaxis, as well as possessing antioxidant properties [12].

The acknowledgment of the science of nutraceuticals has been growing, with increased interest in finding novel therapeutic options by utilizing new technologies and scientific methods. Although many nutraceuticals have been reported for their effective properties in the immune system, there is a necessity of conducting more high-level, clearly evidenced clinical trials for further investigation regarding the long-term effects and public safety. We have observed that it is necessary to broadly exploit the medicinal properties and nutritional values of those compounds separately, because nutraceuticals still lie in the grey zone; being confused by many whether they should be administrated as medicine, or if they are a basic nutrient need. The advanced exploration of their safety, bioactivity, and bioavailability perspective is crucial because it will contribute to translate these hypothetically potential natural nutraceutical compounds into implementable, validated, regulated, and approved effective medicinal products.

The main aim of this review is to highlight recent studies’ outcomes of the immune-boosting properties of nutraceutical compounds and the potential therapeutic activities, including an overview on several types of nutraceuticals and different mechanisms of action on the immune system. Additionally, the safety of nutraceuticals, possible applications, and future prospects will be discussed.

## 2. Types of Nutraceuticals Based on Source, Nature and Application

Nutraceuticals have been classified based on their application into traditional, non-traditional, fortified, recombinant, phytochemical, herbal, functional foods, dietary supplements, probiotics and prebiotics [13,14]. Nutraceuticals with their different classes have a variety of applications and uses depending on their nature. The following subsections will discuss different nutraceutical classes. Table 1 also summarizes the classes of nutraceuticals with their beneficial effects to health.

The classification of nutraceuticals and their definitions tend to overlap due to the similarity among their chemical constituents and functions in delivering health benefits. The Institute of Food Technologists (IFT) defines functional foods as “foods and food components that provide a health benefit beyond basic nutrition”. Examples may include conventional foods; fortified, enriched, or enhanced foods; and dietary supplements [15]. Traditional nutraceuticals are defined as natural foods with their potential health attributes: this may include, but is not limited to, fruits, vegetables, grains, fish, dairy and meat products [16]. Traditional foods or nutraceuticals can positively affect health by stimulating the immune system, and lowering the risk of heart diseases and cancers [17,18,19]. Looking at these definitions, the conclusion we have drawn is that nutraceuticals can be classified into traditional and non-traditional groups. Further subclassification of each will be discussed below, although some may overlap.

### 2.1. Traditional Nutraceuticals and Products

#### 2.1.1. Functional Foods

Functional foods are foods with benefits in health improvement and disease prevention other than only providing nutrients [13,20]. These foods have ingredients that enhance antioxidant and anti-inflammatory activities, which are functional to prevent diseases such as type-2 diabetes [21]. These foods are made available for daily consumption for a specific population with a similar quality of other traditional foods in the market [22,23]. Examples of these functional foods are rice, wheat, kidney beans, soybeans, lentils, chocolate, citrus fruits, nuts, and fermented milk [13,24]. Rice is the first staple food consumed by the majority of populations; its nutritional value is as a source of carbohydrates, containing low levels of fat, salt and sugar, because all types of rice are gluten free and contain resistant starch that helps in the growth of healthy bowel bacteria [25]. Traditional rice varieties in India represent a great origin of minerals and vitamins such as niacin, thiamine, iron, riboflavin, vitamin D, and calcium; in addition, they hold higher fiber and lower amounts of sugar [26]. Wheat is the second staple food consumed across the world: wholegrain wheat is made up of three layers which are the bran, the endosperm, and the germ; wholegrain wheat can be processed to produce wheat bran and wheat germ [27]. Wheat brans represent the most beneficial part of wholegrain wheat due it their fiber content which is believed to play a role in improving gastrointestinal health [28].

Additionally, carrots and broccoli are examples of functional foods due to their active components such as sulforaphane, and lycopene [29]. Although functional foods have various health benefits due to several active ingredients, more studies with scientific evidence are needed to provide these products with health claims in their labels [22,30,31,32]. Some of the active ingredients in functional foods are carotenoids, collagen hydrolysate, dietary fibers, and fatty acids that possess various health benefits such as anti-inflammatory activity and enhance body immunity. In the following subsections, the nature and various health benefits of these functional ingredients will be discussed.

#### 2.1.2. Carotenoids

Carotenoids are natural compounds and sources of pigmentation that accumulate abundantly in plants, fruits and vegetables, and algae. A wide range of carotenoid derivatives are found in the human diet, including α-carotene, β-carotene, β-cryptoxanthin, lutein, lycopene, zeaxanthin, crocetin, fucoxanthin and astaxanthin [33,34]. They are renowned for their wide spectrum of beneficial effects to health, including antioxidant and anti-inflammatory properties [35]. In addition, carotenoids exert health benefits over human vision, cognitive functions, heart functions, cancer prevention, and immune functions [36,37,38]. A study revealed the anti-inflammatory activity of two forms of carotenoids, astaxanthin and β-carotene, where both were found to be able to suppress the inflammation induced by *Helicobacter pylori* by inhibiting the production of reactive oxygen species and diminishing the level of inflammatory mediators being expressed [39].

Carotenoids are also known for their antioxidant activity, which is credited to their chemical structure consisting of a series of conjugated C=C bonds. This structure provides carotenoids with the ability to interact with free radicals and act as effective antioxidants [40]. Although carotenoids exhibit radical scavenging activity, which aids in diseases associated with increased oxidative stress, they also exhibit cyto-genotoxic activity.

#### 2.1.3. Collagen Hydrolysate

Collagen is a primary protein in mammals that can be extracted from bovine connective tissues such as skin, bone, cartilage, and tendons. Collagen extraction is obtained by subjecting it to sources of hot water; this provides a partially hydrolyzed product called gelatin. In order to completely hydrolyze gelatin, a process of enzymatic hydrolysis takes place to produce collagen hydrolysates. Collagen hydrolysates provide various beneficial effects such as antioxidant, anti-aging, antitumor, anti-inflammatory and anti-obesity effects [41,42]. A study has shown the immune-boosting effects of collagen hydrolysates that have been extracted from domestic yak (*Bos grunniens*) bone and its potential in improving the adaptive and innate immunity in mice [43]. Furthermore, a study conducted to investigate the health benefits of collagen hydrolysate in females diagnosed with photoaged skin showed a remarkable improvement in skin hydration, wrinkling, and elasticity [44].

#### 2.1.4. Dietary Fibers

Fibers are plant-based non-starch carbohydrates that are poorly digestible and provide various health benefits, as mentioned in many studies, and can be found naturally in a wide variety of foods including vegetables, fruits, wheat bran, oats and ispaghula husk [45,46,47]. Dietary fibers can be classified into more than two categories on the basis of their solubility in hot water, water-retaining capacity, and viscosity into soluble and insoluble fibers. Soluble fibers comprise viscous components such as β-glucans, fructans, and non-viscous fibers such as hemicellulose. Insoluble fibers tend to lose the characteristic of viscosity and they are insoluble in water; insoluble fibers tend to accelerate gastric emptying time which helps in relieving constipation, while soluble fibers tend to delay gastric emptying time [48]. High fiber diets are found to have a positive impact on inflammatory bowel diseases, because they can lessen the risk of Crohn’s disease and ulcerative colitis [49].

#### 2.1.5. Fatty Acids

Fatty acids are the component of oils and fats that are present in animal fats, fish oil supplements, seeds, olive oil, and coconuts. Aside from their role in energy storage, they have been documented for their ability to act as an anti-inflammatory and immunomodulatory component in various studies. In one study, the omega-3 polyunsaturated fatty acids (PUFAs) administered at a dose of >2.7 g/day for at least three months to patients with rheumatoid arthritis (RA) showed reductions in the severity of rheumatoid arthritis (RA) symptoms [50].

#### 2.1.6. Phytochemicals

Phytochemicals are beneficial, concentrated or purified chemicals from plants that have active components for biochemical and metabolic reactions in humans, such as lutein and lycopene [13]. Phytochemicals can help in maintaining chemical balance of the brain, thus providing neuroprotective activity [51]. Additionally, high consumption of vegetables and fruits that contain phytochemicals can reduce the risk of cancers, and cardiac and neurodegenerative disorders [29,51].

#### 2.1.7. Herbs

Herbs are plants that have no woody tissue and can be processed in many ways depending on each individual preference. Herbs can be dried; however, the drying process leads to a reduction in the effectiveness of herbal properties [52]. Herbs that are rich in antioxidant have been used in flavoring and aroma for more than two thousand years [53]. Garlic extracts, ginger root, and aloe gel are herbs that have health benefits such as reducing cholesterol, wound healing, and anti-ulcer and antioxidant activities [13,54].

#### 2.1.8. Probiotics

Probiotics are microbes that are beneficial to health and used in food, especially in milk products, which are important to promote health by providing immunologic and digestive properties [55]. In addition, these live microbes can improve microbial intestinal balance. *Lactobacillus* with its different species is the most common probiotic used that will survive in the human gut. Currently, *Bifidobacterium* spp., and *Streptococcus* are also used as probiotic strains [56].

#### 2.1.9. Prebiotics

Prebiotics are ingredients consisting of short chain carbohydrates that improve the probiotics’ activity [57]. These prebiotics are literally fertilizing agents for probiotics that are not affected by gastric pH and stomach acids [13]. Prebiotics are non-digestible ingredients that promote the growth of productive microorganism and affect the composition and activity of gut microbiota. Fructo-oligosaccharides and inulin are examples of prebiotics used in functional foods to improve gastric health [57,58].

#### 2.1.10. Dietary Supplements

Although not entirely a traditional approach, dietary supplements are products that can be taken as a dietary ingredient by individuals to maintain and improve health and not to cure diseases [13,59]. These supplements are found in various forms, such as tablets, liquid-based, capsules, powder, and concentrated with specific doses [2,60]. Omega-3, vitamins A, B, C, D, and E, iron, folic acid, minerals, calcium, magnesium, etc., are some examples of dietary supplements that can either be taken by an individual with or without prescription [2,59]. Moreover, these supplements can be consumed to ensure that a diet meets the sufficient nutrient requirements for the body and to prevent any deficiencies [61]. At the beginning of the 20th century, food extracts that contain important nutrients such as vitamin C, and B were shown to be helpful to prevent some serious conditions such as scurvy, pellagra, and beriberi [61,62].

### 2.2. Non-Conventional Approach

Non-traditional nutraceuticals, as a non-conventional approach, are artificially synthesized foods or food products. The application of biotechnology or agriculture breeding is used to add nutrient ingredients for the enhancement of food properties and human health. Based on the processing method, non-traditional nutraceuticals may be differentiated into fortified and recombinant nutraceuticals [14,60,63]. Rice enriched with β-carotene, and cereals infused with vitamins and minerals are some examples of this class of nutraceuticals which contain provitamin A that can boost antioxidant activity [13,64,65].

#### 2.2.1. Fortified Nutraceuticals

Fortified nutraceuticals such as orange juice with calcium added, or milk with cholecalciferol vitamin are foods that contain additional micronutrients or vitamins added to them to improve their value [13,14,60]. These foods supply the body with important nutrients that can prevent anemia and improve health [2,66]. For example, if calcium is added to specific food such as orange juice, the orange juice can enhance glycemic control [67,68].

#### 2.2.2. Recombinant Nutraceuticals

Recombinant nutraceuticals are foods that are produced by both genetic recombination and biotechnology [14]. This type of foods and crops are genetically modified to develop products that contain recombinant compounds and proteins that would be make them more beneficial to health [69]. Iron rice, golden rice, maize, golden mustard, multivitamin corn, and gold kiwifruit are examples of these nutraceuticals. Gold kiwifruit contains a recombinant gene that increases ascorbic acid levels, carotenoid, and lutein to enhance immune function. Additionally, it is considered a source of vitamins, potassium and fiber [70,71,72].

**Table 1:** Summary of types of nutraceuticals and their potential effects on health.

Class/Type of Nutraceutical	Examples	Active Ingredient	Advantages	References
Traditional approaches				
**Functional foods**	Tomatoes	Lycopene	Anticancer activities, e.g., lung and prostate, reduce blood pressure	[17]
	Salmon	Omega 3	Lower cardiovascular, diabetes disease risk	[19]
	Soy	Saponins	Antioxidant, detoxification of enzymes, stimulate immune response, hormonal metabolism	[18]
	Fermented milk and milk products	*L. acidophilus*, *Bifidobacterium* spp.	Prevent gastrointestinal infections, lower the level of cholesterol	[73]
	Marine algae	Fucoidans	Antioxidant, anticancer, anticoagulant activity	[74]
	Broccoli	Sulforaphane, glucosinolates	Decrease risk of several cancers, antioxidant	[29,75]
	Carrots	β-carotene	Reduce cancer risk, improve immune system	[29,76]
	Aloe	Aloins	Wound healing, antiulcer, anti-inflammatory, immunostimulant, antimicrobial activity, hematopoietic stimulation	[77,78]
	Turmeric	Curcumin	Anti-inflammatory, anticarcinogenic	[77,79]
**Dietary supplements**	Folic acid		Prevent defect in neural tubes, Red blood cells formation	[77,80]
	Vitamin A		Antioxidant, growth, treat some skin diseases	[60]
	Calcium		Bone, muscles, teeth nerve health, prevent osteoporosis	[81,82]
	Iron		Carry oxygen, produce energy	[60]
	Vitamin D		Bone and teeth health, help in calcium absorption, musculoskeletal health	[83]
**Probiotics**	*Lactobacillus acidophilus*, *Bifidobacterium* spp., *Streptococci*, *Enterococci*		Gut health, replace diarrhea-causing bacteria, anticancer	[60,84,85]
**Prebiotics**	Fructo-oligosaccharides		Enhance probiotics growth, *bifidobacteria* growth enhancement	[58]
	Inulin		Enhance immune system, minerals absorption, protect bones	[57,84,86]
**Non-conventional approach**				
**Fortified**	Orange juice with calcium	Calcium, ascorbic acid	Glycemic control enhancement, sensitivity to insulin	[67]
	Anthocyanin-fortified bread	Anthocyanin	Reduce digestion rate	[68]
**Recombinant**	Gold kiwifruit	Ascorbic acid, carotenoids	Immune system enhancement	[13,71]

## 3. Classification of Nutraceuticals Based on Modes of Action

It is believed that nutraceuticals enhance human health and increase life expectancy along with many other processes that delay aging and prevent chronic diseases [87]. Many nutraceutical supplements have shown a positive impact on cardiovascular disease, cancer, diabetes, obesity, osteoporosis and immune functions [88,89,90]. Generally, nutraceutical modes of action take place to increase functional components which will lead to health enhancement [91]. This section will discuss various biological activities in nutraceuticals.

### 3.1. Anti-Cancer Activity

The use of nutraceuticals as chemo-preventative agents has been studied, and promising results were obtained as per their ability to prevent and treat cancer. Nutraceuticals of different origins have been shown to exhibit anti-cancer activity. Plants such as garlic, ginseng, curcumin, ginger, and green tea extract express several mechanisms of action against oncogenesis. Such mechanisms include the inhibition of DNA alkylation, tumor initiation, proliferation, and metastasis, in addition to the promotion of autophagy and intrinsic apoptosis [92]. Furthermore, nutraceuticals have been found to attenuate cancer signaling pathways that are believed to play a role in carcinogenesis [93]. Many studies have been conducted to evaluate nutraceuticals’ modes of action against various types of cancer, and a wide range of nutraceuticals have been found to express anti-cancer properties against oral cancer, prostate cancer, breast cancer, lung cancer and colon cancer cells [94,95].

Activation of vitamin D receptor (VDR) results in cell cycle arrest, apoptosis, and anti-angiogenesis [96,97]. This receptor is an intracellular nuclear receptor found in organs and tissues. The active form of vitamin D binds to VDR, leading to activation of the growth arrest gene and *DNA-damage-inducible* gene [96,97]. This concludes that vitamin D deficiency would give rise to many diseases, while sufficient intake of vitamin D aids in disease prevention. In addition, the potentials of vitamins such as vitamin A, C and D have been studied for their mechanisms as anti-cancer agents. On the other hand, complementary therapy incorporating the nutraceutical herb *Clinacanthus nutans* and gemcitabine has been investigated. It has been found that application of *C. nutans* alone or combined with gemcitabine exhibited anti-proliferative effects on pancreatic cancer cells. The summary of this combinatory therapy is the upregulation of Bax and downregulation of Bcl-2, cIAP-2, and XIAP in human pancreatic cancer cells [98].

The role of prebiotics and probiotics has been investigated widely in colorectal cancer (CRC). Both prebiotics and probiotics are believed to be of benefit in promoting human health, specifically in the gastrointestinal tract. A study investigating the effect of prebiotics’ anti-cancer activity showed that high fiber intake elevates the number of short chain fatty acids (SCFAs), thus producing bacteria and resulting in reduced number of colon tumors [99]. Similarly, a study aiming to investigate the prophylactic activity of *Lactobacillus rhamnosus* on the carcinogenesis of CRC showed that oral administration of this probiotic inhibited inflammation associated with tumor development by observing an increase in the expression of inflammatory proteins such as NFκB-p65, TNFα and iNOS [100]. Moreover, tumor incidence, tumor multiplicity, and tumor size are all reduced via consumption of probiotics *Lactobacilus acidophilus*, *Bifidobacteria* spp., and a combination of fructo-oligosaccharide and maltodextrin [101]. Additionally, the synbiotic mechanism lies in facilitating apoptotic responses to carcinogenesis-induced DNA damage in the colon, SCFA production, and downregulation of carcinogenic enzymes [102]. This concludes that the consumption of prebiotics and probiotics exerts immunomodulatory and anti-inflammatory activity and increases lactic acid-producing bacteria, which in return express their anti-cancer activity in colorectal cancer.

Prostate, breast, skin, lung, and liver cancer have been shown to be targeted by polyphenols particularly in green tea as chemo-preventive agents. Green tea is abundant with catechins that possess antioxidant, anti-inflammatory, antiproliferative, and antiangiogenic activity against cancer [103]. A study investigating habitual green tea consumption among human subjects revealed that Epigallocatechin gallate (EGCG) reduced the risk of prostate cancer [104]. Furthermore, the treatment of prostatic carcinoma cell line PC-3 with green tea polyphenol E induced cell death by prompting intracellular oxidative stress that subsequently inhibits the pro-survival pathway Akt [105]. Additionally, the administration of EGCG demonstrated an inhibitory effect on lung cancer cells by activating reactive oxygen species (ROS) that lead to oxidative DNA damage, causing cancerous growth inhibition [106]. Apart from this, a practical study evaluating the effect of EGCG on mammary tumors showed that the administration of poly E resulted in slowing tumor progression, reductions in metastasis, inhibiting mammary ductal growth, and affecting angiogenesis by reducing vascular endothelial growth factor (VEGF) levels. VEGF is an influential angiogenic factor that allows for tumor proliferation and metastatic growth [107]. Additionally, EGCG has shown its potential in inhibiting hepatocellular carcinoma growth by inhibiting the proliferation of HepG2 and PC12, as well as inducing tumor cell apoptosis [108].

Chemopreventive, chemosensitization, and radiosensitization effects have been indicated by curcumin. The curcumin mode of action refers to its ability in inhibiting multiple cell signaling pathways such as AP-1 signaling, NF-κB signaling, and the Wnt/beta-catenin signaling pathway [109]. The inhibition of NF-κB signaling increases cancer cells’ vulnerability to radiotherapy; thus, curcumins’ ability in inhibiting signaling pathways serves as one of its radiosensitizing activity. The administration of curcumin demonstrated the effects on T cell lymphoma, and the results indicate a decline in VEGF proteins resulting in angiogenesis inhibition [110]. In addition, the same study found that curcumin inhibits Glut1, thus resulting in decreased glucose uptake leading to induced apoptosis [110]. An in vitro study on breast cancer cells showed that curcumin decreased cell viability and induced apoptosis by targeting the PI3K signaling pathway [111]. Additionally, it has been found that curcumin up-regulates the expression of miR-99a, which consequently lead to deactivation of the JAK/STAT signaling pathway. The previous processes exerted anti-tumor results such as inhibited proliferation, migration, invasion, and induced apoptosis in retinoblastoma cells [112]. These examples explain curcumin’s activity against tumor progression factors, which justify its chemopreventive effect.

Cell signaling modulation activity has been shown by resveratrol, a polyphenol acting as a chemosensitizer by inhibiting NF-κB and STAT3 pathways [113]. Furthermore, due to resveratrol’s ability in reducing oxidative stress and upregulating the expression of survival proteins, it is used to reduce the cellular damage of chemotherapeutic agents [114]. A study conducted to assess the anti-cancer activity of resveratrol in melanoma determined that resveratrol downregulates NF-κB expression, which in return inhibits the expression of miR-221. The subsequent inhibition process leads to reductions in metastasis and cancer cell proliferation [115]. Additionally, a study revealed that the administration of resveratrol at high concentrations together with long-term treatment led to the promotion of apoptosis and suppression of cancerous cell growth in human non-small cell lung cancer [116]. Overall, nutraceuticals and functional foods participate in cancer treatment as either chemopreventive or chemosensitizing agents. Table 2 summarizes anti-cancer modes of action.

### 3.2. Anti-Inflammatory Activity

Nutraceuticals exert anti-inflammatory activities which help in the prevention and treatment of chronic inflammation-associated diseases [136]. Another benefit of nutraceuticals as anti-inflammatory agents is that they can be used as a complementary alternative to anti-inflammatory therapeutic drugs, which leads to a reduction in drug dosage, and therefore reducing side effects [137]. Chronic inflammation is the major cause of chronic diseases such as cardiovascular diseases, pulmonary diseases, diabetes, and cancer [136].

Suppression of inflammatory cytokines such as interleukins, TNF-α and cyclooxygenase-2 (COX-2) can occur upon the administration of a potent anti-inflammatory such as curcumin [138]. Curcumin can act as an anti-inflammatory agent which can help in the prevention and treatment of periodontitis [139]. Curcumin can reduce inflammation in patients suffering from chronic cutaneous complications and improve patient’s condition suffered from chronic pruritis caused by a chemical sulfur mustard by reducing IL-8 and high-sensitivity c-reactive protein (hs-CRP) [140].

PUFAs are an example of nutraceuticals that have been shown to control inflammatory disorders [141]. Treatment with PUFA reduced the expression of NF-κB. In addition, a reduction in proinflammatory markers and an increase in IL-10 anti-inflammatory marker was observed in patients suffering from Duchenne muscular dystrophy [142]. Another example of a nutraceutical component is lycopene, which is an anti-inflammatory molecule found in tomatoes that can protect the heart and prevent cardiovascular diseases such as atherosclerosis and myocarditis [143,144]. A meta-analysis conducted to evaluate the association between lycopene and cardiovascular disease (CVD) showed a reduction in CVD risk by 17% [145].

Probiotics with anti-inflammatory activity, exert its anti-inflammatory effect through modulation of the NF-κB signaling pathway, inflammatory cytokines, and the regulatory T cell response [146]. For instance, the mixture of probiotics *L. rhamnosus*, *Bifidobacterium lactis*, and *Bifidobacterium longum* exhibited anti-inflammatory activity by inducing IL-10 and reducing proinflammatory cytokine production [147]. Additionally, prebiotics have also been suggested to express anti-inflammatory and immunomodulatory functions [148]. Pretreatment with β-(1,3)-glucan prevented clinical manifestations of dextran sulfate sodium-induced inflammatory bowel disease and inhibited the expression of inflammatory cytokines and reactive oxygen species (ROS) in mice [149].

Anti-inflammatory activities have also been exhibited by ginger, cinnamon, peppermint, and lycopene. Ginger and its compounds exert anti-inflammation activity which can reduce the inflammation [150]. In a study, orally administered ginger to newborn rats with necrotizing enterocolitis showed a reduction in TNF-α, IL-1β, and IL-6, which indicated a significant reduction in the inflammation [150], as well as inhibiting the acute inflammatory response in ulcerative colitis [151]. Furthermore, cinnamon extracts were able to inhibit more than 90% of the expression of IL-1 at the concentration of 50 μg/mL, and peppermint extracts were capable of reducing 90% of the expression of IL-6 at the concentration of 50 μg/mL; both indicated potent levels of anti-inflammatory activity [152]. Table 3 summarizes anti-inflammatory modes of action.

### 3.3. Antioxidant Activity

Oxidative stress is a result of the accumulation of free radicals in the body which can subsequently lead to the development of various chronic diseases such as cancer, cardiovascular and autoimmune disease, ischemic disease, atherosclerosis, diabetes mellitus, and hypertension [163,164]. Normally, redox (reduction and oxidation) balance of the cell maintains the generation and the removal of reactive oxygen species (ROS) [165]. However, an imbalance in redox will result in the accumulation of ROS and reducing antioxidant ability to neutralize ROS effects, which subsequently induces oxidative stress [166,167].

Exogenous antioxidants such as vitamin C, vitamin E, and phenolic antioxidants are capable of removing free radicals [168]. Free radicals such as hydroxyl radicals and superoxide anion radicals can be scavenged by vitamin C [168]. Vitamin C is a potent antioxidant that protects cells and DNA from oxidative stress by scavenging free radicals [169]. Vitamin E and vitamin C are capable of protecting cells from lipid peroxidation [170].

Ginger extract and quercetin are also antioxidants; however, the ability of ginger extract to inhibit hydroxyl radicals is greater than quercetin [171]. Ginger extract showed an increase in the antioxidant enzyme and a reduction in the oxidative stress in blood [172].

The main sources of antioxidants are food, vitamins, and supplements. Foods such as fruits and vegetables are considered a great source of antioxidants due to their high levels of vitamins and phytochemicals [173]. Beetroot contains betalain and phenolic compounds which cause an increase in the resistance of low-density lipoproteins (LDLs) to oxidation [174], protect the liver from damage [175], and decrease blood pressure [176]. Dried fruits are a good source of antioxidants and have health benefits to humans. They can reduce glucose levels in the blood in addition to reducing risk factors associated with heart disease [177].

Dates are an example of dried fruit which contains two compounds, isoflavones and lignans; these two compounds can act as antioxidants which have a role in diabetes and are capable of modulating the secretion of pancreatic insulin [178]. Nuts such as pistachios are a rich source of antioxidants [179] which can reduce oxidative stress and prevent or lower the risk of chronic diseases [180]. Pistachios and other nuts such as walnuts and pecans contain polyphenolic compounds which act as antioxidants [181] that might protect against diseases associated with the accumulation of free radicals [180].

### 3.4. Anti-Lipid Activity

Nutraceuticals such as vitamins, minerals, and antioxidants are considered useful in the management of hypercholesterolemia in many conditions such as hypertension, diabetes, and cardiovascular disease [88,182,183]. Hypercholesterolemia is a term used to describe the presence of excessive low-density lipoproteins in blood [184]. The effects of nutraceuticals on lowering lipid profiles in hypercholesterolemic patients have been investigated; therefore, this section will discuss nutraceuticals’ activity on multiple conditions associated with elevated lipid levels. The application of nutraceuticals as hypolipidemic agents has shown great potential in lowering total cholesterol (TC) and low-density lipoprotein (LDL) concentrations. Lipid-lowering nutraceuticals can be classified into three groups based on their mechanism of action. Such mechanisms include the inhibition of cholesterol absorption, inhibition of cholesterol synthesis, and excretion of LDL [185].

Plant sterol foods or supplements have displayed effectiveness in lowering lipid profiles. It has been established that plant sterols modify lipid profiles by decreasing the intestinal absorption of cholesterol [186]. It has been found that the intake of plant sterols demonstrated an effect on lipid profiles by lowering the concentrations of triglycerides and LDL in individuals at risk or suffering from type-2 diabetes mellitus [187]. A study examining the effectiveness of plant sterol-enriched yogurt has demonstrated a modified lipid profile resulting in reduced TC and LDL concentrations [188]. Moreover, the additive effect of plant sterols with lipid-lowering therapies have been investigated, and it has been determined that the addition of plant sterols to atorvastatin or ezetimibe resulted in further reductions in total and low density cholesterol [189].

Red yeast rice (RYR) exhibited potent effects on lowering lipid profiles in patients suffering from cardiovascular disease and dyslipidemia [190,191,192]. A study was conducted to assess a group of nutraceuticals on their lipid modifying ability; the nutraceuticals of choice were berberine, RYR and monacolin K, policosanol, and folic acid. The results demonstrated that RYR significantly lowered TC and LDL [183]. The combination of fermented red rice, liposomal berberine, and curcumin showed great results in improving lipid profiles and reducing inflammatory markers in individuals suffering from hypercholesterolemia [193]. RYR possess many functional components, although monacolin K may be a major contributor to its effectiveness. Monacolin K mechanism of action lies in decreasing hepatic cholesterol synthesis and inhibiting cholesterol absorption [194].

The consumption of dietary fibers is considered an effective approach in modulating lipid profiles. Dietary fibers are divided into soluble and insoluble fibers. Soluble fibers are more beneficial because they are fermented by the microbiota of the large intestine [195].

Cholesterol-lowering activity has been demonstrated by berberine, a plant alkaloid known for its cholesterol-lowering activity [196]. Similarly, curcumin is also considered a hypolipidemic agent that reduces TC, LDL and triglycerides, which is explained by increased cholesterol excretion [197].

In summary, many nutraceuticals and functional foods have been proven for their ability in regulating lipid profiles. Owing to their mechanism of action, nutraceuticals can either inhibit cholesterol synthesis, inhibit cholesterol absorption, or enhance cholesterol excretion. Each nutraceutical mentioned above may display a single or combined mechanism of action, resulting in lowered TC and LDL.

## 4. Nutraceuticals’ Safety on Consumers

The majority of nutraceuticals on the market are safe for human consumption and only in some instances may cause harm because some nutraceuticals have toxic effects on human health. Studies have shown that some widely consumed nutraceuticals possess many health benefits with very minimal toxicity when used in correct controllable amounts. Nutraceuticals such as anthocyanins, polyphenols, and catechins are widely used and are safe for human consumption on controlled use. There have been very few studies that have indicated how these substances are harmful to human health.

Nevertheless, studies on nutraceuticals have shown that benefits of use outweigh the risk, and they are widely approved for human use within the correct amounts and dosages [185]. However, misuse and overuse of these products may pose health risks to humans. The safety of these nutraceuticals on consumers basically depends on the type, time, and the quantity used. Use of some nutraceuticals, especially when one is under medication, can result in interactions between the drug and the nutraceutical compounds causing very harmful effects on the body [198]. Thus, for safe usage, they should be used only when prescribed by qualified personnel at the right quantity, the correct quality, and timing.

Among the most important features of nutraceuticals are their cost effectiveness, broad safety profiles both in humans and animals, tolerability, and easy availability [199]. Despite a broad safety profile, few of them are reported to have been compromised due to contamination with heavy metals, toxic pesticides, drugs having potentials for abuse, potentially toxic plants, fertilizers, and mycotoxins [200,201]. Unfortunately, the safety profile of a large number of nutraceuticals is yet to be explored, and thus insufficient safety data are available for these agents. Understanding of the pharmacokinetic behavior of every drug is extremely important for the understanding of safety profile (toxico-kinetics), onset of action, required dose, and dose frequency. Furthermore, interactions with other drugs as well as nutrients/foods are another vital aspect of safety evaluations of nutraceuticals that assess the effect of interactions on safety, efficacy, half-life and subsequent therapeutic response [198].

In a majority of countries, including the United States, nutraceuticals are included in the dietary supplements list and may not be subject to the laws and regulations for safety standards of allopathic drugs. In 1994, the U.S. Congress established a regulation called the “Dietary Supplements Health & Education Act” which included nutraceuticals in a dietary list. These are not considered drugs; therefore, their sale without any safety and efficacy evaluation is permitted. However, as per regulations of the European Union, these herbal agents must prove scientific evidence of safety, efficacy, and quality before being licensed for use in the public. Toxicity and therapeutic evaluations of nutraceuticals are difficult as compared to pure synthetic compound-based products because they have multiple compounds and usually are a complex mixture of several compounds. Furthermore, their chemical composition varies based on the area of plant collection, variable effects of fertilizers/pesticides, and effect of stress [202]. All of the above factors, in addition to the unavailability of well-established techniques for extraction, identification, chemical composition, purity, potency, and safety of active pharmaceutical ingredients are the limiting factors of nutraceuticals, responsible for batch to batch variation and the un-reproducibility of therapeutic responses [203]. Furthermore, nutraceuticals comprise numerous compounds which can act synergistically as well as mediate an antagonistic response. Both of these effects may range from high therapeutic outcomes to toxicity and possibly a sub-therapeutic response [204].

In short, the use of nutraceuticals is tremendously increased both in humans as well as in veterinary products. Consequently, toxicity risks associated with their use are also increased due to the unavailability of large-scale evaluations of their safety and efficacy in clinical trials. Furthermore, controlling variations and the presence of adulterants such as heavy metals, agricultural chemicals, and mycotoxins, as well as proper pharmacokinetic and dynamic evaluations are extremely necessary for more effective utilization of these important alternative agents.

### 4.1. Nutraceuticals Associated with Genotoxicity and Carcinogenicity

Nutraceutical-associated genotoxicity is the potential of a nutraceutical product or its component to cause DNA lesion resulting in cellular death or genetic mutations, which may lead to various types of cancers. However, *Aloe vera* is recommended as a remedy for many diseases; studies involving oral therapy with *Aloe vera* whole leaf extract for two years in F344/N rats evidenced some carcinogenic effects [205,206,207,208]. *Aloe vera* gel has been generally found to be less genotoxic and less mutagenic as compared to whole leaf extracts [209], and in agreement, it was unable to induce significant mutagenic effects in *S. typhimurium* TA100 strain [210]. However, a positive mutagenic response was observed when an *E. coli* repair-deficient mutant was treated with *A. vera* pulp extract [211]. Danthron, an aloe constituent, is reported to initiate DNA damage and cause caspase-induced apoptosis via Bax-elicited and mitochondrial permeability transition pore pathways in human gastric cancer cells [212].

In a different study, mutagenic effects were observed via the development of hepatocellular carcinoma and hepa-blastoma in B6C3F1 mice in the presence and absence of external S9 (liver extract from hamsters and rats) metabolic activation after chronic administration of *Ginkgo biloba*. Mutagenic effects in *S. typhimurium* strains TA98 and TA, and *E. coli* WP2 *uvrA* pKM101 strain were also observed [213]. Several in-vitro studies using cell lines demonstrated *G. biloba* as well as its isolated components including quercetin, kaempferol-induced cytotoxicity, and mutagenicity [214]. However, no genotoxicity was experienced in androstane receptor knockout and *gpt* delta mice following oral administration of 2000 mg/kg *G. biloba* extract [215,216]. Therefore, testing against *S. typhimurium* (TA98, TA100 strains) and *E. coli* (WP2 *uvrA*/pKM101 strain) revealed that 1–10 mg/plate of goldenseal root powder was not mutagenic, while hepatocellular carcinoma/adenoma were observed in F344/N rats and B6C3F1 mice orally supplemented with the same nutraceutical for two years. However, when supplementation was performed only for three months, increases in micro-nucleated erythrocyte frequencies were not significant [217]. In vitro studies on HepG2 cells using commercial goldenseal products suggested that it damages DNA, indicated by the induction of gamma-H2AX, which is a biomarker of DNA damage and breaks [218].

Consumption of herbal products has been tremendously increased worldwide despite inconsistencies in scientific evidence, although their health promoting benefits are reported by numerous studies. Results of these studies suggest that despite dietary supplements, herbs, and nutraceuticals are generally considered safe, however there is still a need for rigorous toxicological testing. The currently available data regarding genotoxic and mutagenic effects of these products are insufficient to provide conclusive outcomes of the safe use of all nutraceuticals.

### 4.2. Models to Evaluate Safety, Efficacy, and Potential Toxicities of Nutraceuticals

Several models have been developed to evaluate the safety, efficacy, and toxicity of nutraceutical products and their active ingredients. Bioactive ingredients present in nutraceuticals have exhibited diverse pharmacological properties such as antioxidant, sedative, hypnotic, anti-inflammatory, immuno-modulatory and adaptogenic attributes [219,220,221,222,223]. Subsequently, these products were applied and their efficiency appraised using in vitro, in vivo, in silico, high-throughput in vitro, and omics technology-reliant assays [224]. Furthermore, various animal models have been used for in vivo, invasive, and non-invasive studies depending upon study design [225]. Some invertebrate models have been developed for safety and efficacy evaluations of nutraceuticals [226,227]. Currently, some alternative safety evaluation models are more preferred [228]. Novel and mechanism-based predictive kinetic models have also been developed, which provide very useful information regarding pathways involved in toxicity and efficacy [229,230].

### 4.3. Toxicities Based on Interactions of Nutraceuticals with Other Drugs

Interaction of a drug with any herbal or dietary supplement may lead to severe undesirable health consequences [231]. Several factors including age, simultaneously administered drugs, and multiple co-morbidities such as hypertension, diabetes, cancer, and infectious diseases contribute to these unwanted interactions and pre-dispose patients to the side effects of nutraceuticals. Of particular importance are drugs with a low therapeutic index such as digitalis glycosides, anticoagulant drugs, chemotherapeutics, psychoactive agents, and immune-modulators which can lead to life threatening reactions if their concentrations rise beyond the safety range due to interactions [231]. Some nutraceuticals are inducers and inhibitors of cytochrome P450 (CYTP450) iso-enzymes, and thus induce or inhibit liver enzymes responsible for the metabolism of these drugs [232,233,234]. For instance, several phytochemicals present in *G. biloba* are inducers as well as inhibitors of CYP450 [235,236]. Ginseng is an inducer of CYP450, whereas grapefruit juice is an inhibitor of it. Likewise, peppermint oil is a well-studied inducer of several CYP450 isoforms including CYP1A2, CYP2C9, CYP2C19 and CYP3A4 [237]. Furthermore, St. John’s wort and its constituents inhibit CYP3A4-induced metabolism of testosterone and midazolam. Studies revealed that several fold increases in the metabolic activity of hepatic CYP3A2 were observed fourteen days subsequently of St. John’s wort administration [238,239]. Moreover, ginseng, an inhibitor of P-glycoproteins type efflux pumps, causes a 3–4-fold increase in the activity of several drugs [238]. St. John’s wort administration has major effects on the kinetics and dynamics of several drugs, especially when poly-pharmacy is involved [240,241]. Most types of drug may affect nutriture, directly or indirectly, whereas nutriture affects drug disposition and subsequent therapeutic responses.

### 4.4. Contaminants Compromising the Quality of Nutraceuticals

Several toxic adulterants or contaminants such as heavy metals, pesticides, mycotoxins, phytotoxicants, and abused drugs can significantly compromise the quality as well as use of nutraceuticals. Mixing of these adulterants in high amounts can lead to severe health consequences and even death [242]. For instance, pyrrolizidine alkaloids are among the most toxic alkaloids present in several plant species. These alkaloids contaminate several foods and nutraceuticals and react with proteins causing abnormal mitosis, tissue necrosis, and cellular dysfunctions [243,244,245]. These are hepatotoxic and have carcinogenic and cytotoxic potentials [242].

On the other hand, microbial contamination may contribute to a deterioration in the quality and stability of dietary foods and supplements [246]. For example, a Polish study showed that from 152 products and samples tested, 92.1% and 86.8% exhibited different degrees of bacterial and fungal contamination, respectively [246].

Mycotoxins are fungal secondary metabolites which significantly affect food and nutraceutical quality. These are usually unintentionally added to the foods/products during cultivation, storage, and transportation. Some common mycotoxins include citrinin, aflatoxins, ochratoxins and other molds and their spores [247,248]. Likewise, heavy metals including mercury, arsenic, cadmium and lead are contaminates added during harvesting or storage; therefore, the World Health Organization (WHO) strongly recommends to evaluate all herbal products for heavy metal contents due to their severe side effects [249]. Similarly, widespread use of pesticides causes accumulation in the products and causes toxicity [250].

### 4.5. Regulatory Status of Nutraceuticals

Several organizations including government agencies have promoted nutraceuticals and functional foods, and the public awareness regarding the use of these products has been increased. In several countries, no specific standard guidelines are available regarding the production and use of nutraceuticals. In the United States, some guidelines are provided in the Dietary Supplement Health and Education Act (DSHEA), adopted by congress for implementation in 1994. Likewise, in Poland, the Food and Nutrition Safety Act was adopted in 2006, whereas Canada has framed specific guidelines in their Food and Drugs Act [16,251]. Proper regulatory recognition of these products will excel the pharmaceutical industry with many new products and subsequent expansion of its market. A majority of countries have not adopted specific regulatory guidelines for nutraceuticals, which could possibly cause flooding of the healthcare sector with low-quality products, especially in developing countries. Thus, there is a dire need for the global health community to prepare regulatory guidelines for nutraceuticals to protect the market from the entry of spurious and low-quality drugs. Furthermore, rigorous scientific research is necessary both in the field of functional foods and nutraceuticals to uncover their exact mechanisms of action as well as potential adverse effects.

### 4.6. Effects of Processing on Nutraceuticals

Previous work considering some post-harvest abiotic elicitors proposed nonthermal processing technologies (i.e., ultrasound, high pressure processing (HPP), and pulsed electric fields (PEF)) to enhance the production of secondary metabolites. Interestingly, however, the outcome of these effects have been shown to be highly similar to conditions of plants under duress [252]. These treatments activate the biosynthesis of nutraceuticals in crops by a similar mechanism exerted by wound stress. Another study has shown that ultrasound treatment increases the levels of carotenoids in carrots [253]. Furthermore, the same treatment enhanced phenylalanine ammonia-lyase activity (PAL) and phenolic compounds in Panax ginseng [254], while phenolic compounds were increased in romaine lettuce [255]. A different study has shown that HHP increased ascorbic acid, phenolic compounds, and carotenoids in ataulfo mango [256]. Additionally, potato metabolism showed PEF-specific responses characterized by changes in the hexose pool, which may result in starch and ascorbic acid degradation [257]. The inadvertent production of metabolites may further enhance the exerted health benefits of such foods compared to foods which are consumed directly post-harvest. However, this is an area that is still under research, and the perceived benefits in the long term to human health are very much unexplored.

## 5. Current Trends and Future Prospects of Nutraceuticals

Over the last 20 years, there has been a rapid increase in the use of nutraceuticals due to mass information available on internet sources coupled with increased public awareness of health issues [258]. The marked side effects and ineffectiveness of modern pharmaceuticals have compelled people to look for nutraceuticals as alternative therapy [259]. Nutraceuticals for a medicinal use have been justified on the basis that they treat disease caused by the deficiency of nutrients. Clear evidence has been reported that nutraceutical supplementation improves health and prevents diseases [260]. The treatment through nutraceutical supplementation does not involve diagnosis by a trained practitioner, and nutraceuticals with antioxidant activity are expected to have beneficial effects on the whole body rather than to treat symptoms of a disease state. Consumers of nutraceuticals control their health comfortably without needing consultation with their physicians.

Self-medications with nutraceutical for the long term may result in cost implications to the consumers and may be more expensive as compared to other medications, despite their benefits [261]. This is due to glorification of the benefits of nutraceuticals via advertising and media coverage. Health professionals such as general physicians, nurses, pharmacists, and nutritionist are well aware of nutraceuticals, and educate their patients or consumers about the appropriate use of such products. Self-medication of nutraceuticals for serious diseases is inappropriate, while their long-term use is safe and beneficial for the prevention of chronic diseases. Nutraceuticals for serious diseases involve carnitine and flaxseed oil mostly used for cardiovascular disease, and antioxidants mostly for the prevention of cancer. Currently, many nutraceutical consumers believe that dietary supplements may be safer than other synthetic substances, but their presumption could be wrong and medical diagnosis is required for serious disease to prescribe effective conventional medicines. It has been reported that self-medication with complementary medicine has increased in diabetic patients [262,263].

Manufacturers of nutraceuticals are well informed about the production cost and profit. Manufacturers of nutraceuticals are also frequently launching new products into the market to expand the nutraceutical industry. The use of nutraceuticals has been encouraged for the prevention and treatment of chronic diseases. For example, green tea and soy products are used to prevent cancer [264].

The developments of new medicines are more expensive and riskier than the already available drugs in the market; therefore, most pharmaceutical companies are turning to marketing nutraceuticals. The drug company “Novartis” has also launched functional food for the health of consumer in the market and pharmacies [265]. In October 1998, Dean Farms, Tring and Hertfordshire introduced “Columbus healthier eggs” which were rich in fatty acid and available in all major supermarkets [266]. Similarly, burgeon bread is another example of function food, which was introduced by Allied Bakeries in September 1997. This bread contains soya and flaxseed and is rich in natural plant estrogen and is used to treat menopausal symptoms [266,267,268].

Meanwhile, government sponsorship for clinical trial has grown, and funding for nutraceutical research has increased. The supply of nutraceuticals and writing of analytical monographs of nutraceuticals for routine quality assurance is controlled by the regulatory authorities. Analytical profiles of products published by consumer organizations enables the consumer to pick out the best quality products. Available data showing the use of nutraceuticals against certain life-threatening diseases are still insufficient to prove the use of nutraceuticals for which they are sold in the market. Therefore, government support is mandatory to improve or strengthen the research in these areas.

The identification of single nucleotide polymorphisms among the human population has enabled us to predict variations in individual responses to drugs and materialize the new concept of personalized medicines. Subsequently nutritional genomics has emerged, which includes dietary component interactions with genomes and results in proteomic and metabolic changes called “nutrigenomics”. Additionally, understanding of genetic differences among individuals has developed; people respond differently to the same nutraceutical, which is called nutrigenetics. The availability of genomic information accelerates the progress of disease treatment, and genotyping is used by the pharmaceutical companies to predict the efficacy, safety, and toxicity of drugs during clinical trials. In pharmacogenomics, the patient’s response to medication is studied, whereas to study the effect of nutraceuticals and dietary components on the health of particular individual, “nutrigenomics” has been developed. Nutrigenomics uses genetic information of a particular individual to predict nutraceutical supplementation for to maintain health or prevent diseases.

## 6. Conclusions

Nutraceuticals embody a novel and exhilarating research field for the discovery of innovative health products with tremendous potentials of health benefits including safety, efficacy, and economy. Globally, researchers have realized the fact that proper nutrition and dietary supplements can prevent and cure chronic diseases. Several types of nutraceuticals have been isolated from foods, and massive quantities are produced using biotechnology and genetic engineering tools which provide pharmaco-economic benefits. These products, beside their nutritional aspects, provide tremendous health benefits via the prevention of several diseases. Nutraceuticals have proven efficacy in numerous diseases, including cancer, rheumatism, diabetes, and other chronic diseases. The use of scientifically and medically approved nutraceuticals can definitely improve health and prevent certain diseases, and some have exhibited the same efficacy as that of conventional pharmaceuticals. Generally, nutraceuticals have lower incidences of side effects, adverse effects, and drug interactions as compared to both complementary medicines and conventional pharmaceuticals. However, risk-benefit uses of nutraceuticals have not yet been documented as well as for other conventional pharmaceuticals, and the absence of side effects, adverse effects, and drug interactions does not indicate that nutraceuticals lack these properties.

This research area has major attractions both for academia and pharmaceutical/food industries. A few pharma-industries including Ranbaxy and Abbot have taken the initiative of synthesizing a range of nutraceutical products for different age consumers. The preventive role of these products is uncovered by researchers to a great extent; therefore, further extensive research both from academia as well as the pharmaceutical sector is necessary regarding their safety and efficacy. Furthermore, use of advanced and high-throughput technologies can help us understand the underlying mechanisms of action and develop this exciting area of research to new horizons for the betterment of humanity, both in terms of economic benefits as well as health outcomes.

## Figures and Tables

**Table 2:** Selected anti-cancer modes of action classified based on cancer type and nutraceutical.

Type of Cancer	Mode of Action	Nutraceutical	References
**Prostate cancer**	Antiproliferation, cell cycle inhibition, angiogenesis inhibition and promotion of apoptosis	Vitamin D	[117]
	Antioxidation, antiproliferation, and promotion of apoptosis	Catechins in green tea	[118,119]
**Colon cancer**	Tumor marker suppression, promotion of apoptosis, metastasis inhibition, and antiproliferation	Polyphenols	[120,121]
	Antioxidant, antiproliferation, promotion of apoptosis, inflammatory protein inhibition	Terpenoids	[122,123]
	Autophagy induction and promotion of apoptosis	Alkaloids	[124,125,126]
	Induction of DNA hypomethylation, promotion of apoptosis, and antiproliferation	Micronutrients	[127,128]
**Breast cancer**	Antiproliferation, angiogenesis inhibition, and promotion of apoptosis	Allicin in garlic	[129,130,131]
	Antiproliferation and promotion of apoptosis	Curcumin	[132]
	Cell cycle inhibition, promotion of apoptosis, and inhibition of metastasis	Vitamin D	[133,134]
**Oral cancer**	Prevent tumor initiation	Strawberry	[127]
	Antioxidation	Rosemary	[128]
	Antiproliferation, promotion of apoptosis, and angiogenesis inhibition	Geraniol	[135]

**Table 3:** Selected anti-inflammatory modes of action classified based on nutraceutical and benefits.

Mode of Action	Nutraceutical	Benefits	Reference
Reduce nitric oxide synthase (iNOS), tumor necrosis factor-α (TNF-α), production of nitric oxide (NO), interleukin-1β (IL-1β), nuclear factor kappa B (NF-κB)	Resveratrol	Neuroprotective	[153]
Inhibit the activation of NF-κB and limits the inflammatory response, such as ICAM-1, MCP-1, Cox-2, TNF-α, IL-1β, and IL-6	Baicalin	Improvement of trinitrobenzene sulphonic acid (TNBS) induced colitis	[154]
Reduce the expression of TNF-α, COX-2, 5-LOX, and IL-6 and increase IL-10 levels	Flavocoxid	Protects from sepsis	[155]
Reduces the expression of TNFα, IL-1β and reduces myeloperoxidase (MPO) activity	Curcumin	Improve dextran sulfate sodium (DSS)-induced colitis	[156]
Decreases the expression of iNOS and COX-2	6-Gingerol	Protects from carbon tetrachloride (CCl4)-induced liver fibrosis	[157]
Inhibits the expression of TLR4 and NF-κB and suppress iNOS, COX-2, TNF-α, IL-6, and IL-1β	Apigenin	Protects against blood-brain barrier disruption	[158]
Reduced the expression of TNF-α, IL-1β, and IL-6 and increases IL-10 expression. Decreases TLR-2 and TLR-4 expression Inhibits phosphorylation of I-κB, p65, p38, ERK, and JNK	Piperine	Reduces inflammatory injury in *Staphylococcus aureus* endometritis.	[159]
Suppresses the activity of renal MPO	Naringin	Decreases neutrophil infiltration in the kidneys.	[160]
Reduces NF-kappa B p65 subunit activation which decreases inflammatory cells and reduces cytokine secretion	Eucalyptol	Potential agent in the treatment of cigarette smoke-induced acute lung inflammation.	[161]
Suppresses NF-kB and p38 and reduces the level of TNF-α and IL-1β levels	Ortho-eugenol	Treatment of pain and inflammation.	[162]

## Data Availability

Not applicant.

## References

[1] Misra L. (2013). Traditional Phytomedicinal Systems, Scientific Validations and Current Popularity as Nutraceuticals. https://www.semanticscholar.org/paper/Traditional-Phytomedicinal-Systems%2C-Scientific-and-Misra/7df8a6c6cc432a4cd711b8b6a96702f1908353d4.

[2] Helal N.A., Eassa H.A., Amer A.M., Eltokhy M.A., Edafiogho I., Nounou M.I. (2019). Nutraceuticals’ Novel Formulations: The Good, the Bad, the Unknown and Patents Involved. Recent Pat. Drug Deliv. Formul..

[3] Petrovska B.B. (2012). Historical review of medicinal plants’ usage. Pharmacogn. Rev..

[4] Nasri H., Baradaran A., Shirzad H., Rafieian-Kopaei M. (2014). New Concepts in Nutraceuticals as Alternative for Pharmaceuticals. Int. J. Prev. Med..

[5] Caramia G., Silvi S., Malago J.J., Koninkx J.F.J.G., Marinsek-Logar R. (2011). Probiotics: From the Ancient Wisdom to the Actual Therapeutical and Nutraceutical Perspective. Probiotic Bacteria and Enteric Infections: Cytoprotection by Probiotic Bacteria.

[6] Ried K. (2016). Garlic Lowers Blood Pressure in Hypertensive Individuals, Regulates Serum Cholesterol, and Stimulates Immunity: An Updated Meta-analysis and Review. J. Nutr..

[7] Affuso F., Ruvolo A., Micillo F., Saccà L., Fazio S. (2010). Effects of a nutraceutical combination (berberine, red yeast rice and policosanols) on lipid levels and endothelial function randomized, double-blind, placebo-controlled study. Nutr. Metab. Cardiovasc. Dis..

[8] Chen G.-L., Chen S.-G., Chen F., Xie Y.-Q., Han M.-D., Luo C.-X., Zhao Y.-Y., Gaob Y.-Q. (2016). Nutraceutical potential and antioxidant benefits of selected fruit seeds subjected to an in vitro digestion. J. Funct. Foods.

[9] Pitchaiah G., Akula A., Chandi V. (2017). Anticancer Potential of Nutraceutical Formulations in MNU-induced Mammary Cancer in Sprague Dawley Rats. Pharmacogn. Mag..

[10] Singla V., Pratap Mouli V., Garg S.K., Rai T., Choudhury B.N., Verma P., Deb R., Tiwari V., Rohatgi S., Dhingra R. (2014). Induction with NCB-02 (curcumin) enema for mild-to-moderate distal ulcerative colitis—A randomized, placebo-controlled, pilot study. J. Crohn’s Colitis.

[11] Chaplin D.D. (2010). Overview of the Immune Response. J. Allergy Clin. Immunol..

[12] Carr A.C., Maggini S. (2017). Vitamin C and Immune Function. Nutrients.

[13] Ruchi S. (2017). Role of nutraceuticals in health care: A review. Int. J. Green Pharm..

[14] Singh J., Sinha S. (2012). Classification, regulatory acts and applications of nutraceuticals for health. Int. J. Pharm. Bio Sci..

[15] Scrinis G. (2008). Functional foods or functionally marketed foods? A critique of, and alternatives to, the category of “functional foods”. Public Health Nutr..

[16] Prabu S.L., SuriyaPrakash T.N.K., Kumar C.D., SureshKuma S., Ragavendran T. (2012). Nutraceuticals: A review. Elixir Int. J..

[17] Bhowmik D., Kumar K.P.S., Paswan S., Srivastava S. (2012). Tomato-A Natural Medicine and Its Health Benefits. J. Pharmacogn. Phytochem..

[18] Singh B., Singh J.P., Kaur A. (2017). Saponins in pulses and their health promoting activities: A review. Food Chem..

[19] Smith L.K., Guentzel L.J. (2010). Mercury concentrations and omega-3 fatty acids in fish and shrimp: Preferential consumption for maximum health benefits. Mar. Pollut. Bull..

[20] Heldman D.R. (1994). Food Science Text Series. http://www.springer.com/series/5999.

[21] Alkhatib A., Tsang C., Tiss A., Bahorun T., Arefanian H., Barake R., Khadir A., Tuomilehto J. (2017). Functional Foods and Lifestyle Approaches for Diabetes Prevention and Management. Nutrients.

[22] Lau T.-C., Chan M.-W., Tan H.-P., Kwek C.-L. (2012). Functional Food: A Growing Trend among the Health Conscious. Asian Soc. Sci..

[23] Smith J., Charter E. (2011). Functional Food Product Development.

[24] Sikand G., Kris-Etherton P., Boulos N.M. (2015). Impact of functional foods on prevention of cardiovascular disease and diabetes. Curr. Cardiol. Rep..

[25] Umadevi M., Pushpa R., Sampathkumar K., Bhowmik D. (2012). Rice-Traditional Medicinal Plant in India. J. Pharmacogn. Phytochem..

[26] Bhat F.M., Riar C.S. (2015). Health Benefits of Traditional Rice Varieties of Temperate Regions. Med. Aromat. Plants.

[27] Stevenson L., Phillips F., O’Sullivan K., Walton J. (2012). Wheat bran: Its composition and benefits to health, a European perspective. Int. J. Food Sci. Nutr..

[28] Prückler M., Siebenhandl-Ehn S., Apprich S., Höltinger S., Haas C., Schmid E., Kneifel W. (2014). Wheat bran-based biorefinery 1: Composition of wheat bran and strategies of functionalization. LWT Food Sci. Technol..

[29] Lobo V., Patil A., Phatak A., Chandra N. (2010). Free radicals, antioxidants and functional foods: Impact on human health. Pharmacogn. Rev..

[30] El Sohaimy S.A. (2012). Functional foods and nutraceuticals-modern approach to food science. World Appl. Sci. J..

[31] Hasler C.M. (2002). Functional foods: Benefits, concerns and challenges-a position paper from the american council on science and health. J Nutr..

[32] Wildman R.E.C., Bruno R.S. (2019). Handbook of Nutraceuticals and Functional Foods.

[33] Lee Y., Hu S., Park Y.-K., Lee J.-Y. (2019). Health Benefits of Carotenoids: A Role of Carotenoids in the Prevention of Non-Alcoholic Fatty Liver Disease. Prev. Nutr. Food Sci..

[34] Shardell M.D., Alley D.E., Hicks G.E., El-Kamary S.S., Miller R.R., Semba R.D., Ferrucci L. (2011). Low-serum carotenoid concentrations and carotenoid interactions predict mortality in US adults: The Third National Health and Nutrition Examination Survey. Nutr Res..

[35] Eggersdorfer M., Wyss A. (2018). Carotenoids in human nutrition and health. Arch. Biochem. Biophys..

[36] Cheng H.M., Koutsidis G., Lodge J.K., Ashor A.W., Siervo M., Lara J. (2019). Lycopene and tomato and risk of cardiovascular diseases: A systematic review and meta-analysis of epidemiological evidence. Crit. Rev. Food Sci. Nutr..

[37] Chew E., Clemons T., SanGiovanni J.P., Denis R., Ferris I.I.I.F., Elman M., Antoszyk A.N., Ruby A.J., Orth D., Bressler S.B. (2014). Secondary Analyses of the Effects of Lutein/Zeaxanthin on Age-Related Macular Degeneration Progression. JAMA Ophthalmol..

[38] Walk A.M., Khan N.A., Barnett S.M., Raine L.B., Kramer A.F., Cohen N.J., Moulton C.J., Renzi-Hammond L.M., Hammond B.R., Hillman C.H. (2017). From neuro-pigments to neural efficiency: The relationship between retinal carotenoids and behavioral and neuroelectric indices of cognitive control in childhood. Int. J. Psychophysiol..

[39] Kang H., Kim H. (2017). Astaxanthin and β-carotene in Helicobacter pylori-induced Gastric Inflammation: A Mini-review on Action Mechanisms. J. Cancer Prev..

[40] Young A.J., Lowe G.L. (2018). Carotenoids-Antioxidant Properties. Antioxidants.

[41] Gómez-Guillén M.C., Giménez B., López-Caballero M.E., Montero M.P. (2011). Functional and bioactive properties of collagen and gelatin from alternative sources: A review. Food Hydrocoll..

[42] Song H., Li B. (2017). Beneficial Effects of Collagen Hydrolysate: A Review on Recent Developments. J. Sci. Tech. Res..

[43] Fan J., Zhuang Y., Li B. (2013). Effects of Collagen and Collagen Hydrolysate from Jellyfish Umbrella on Histological and Immunity Changes of Mice Photoaging. Nutrients.

[44] Do-Un K., Chung H.-C., Choi J., Sakai Y., Boo-Yong L. (2018). Oral Intake of Low-Molecular-Weight Collagen Peptide Improves Hydration, Elasticity, and Wrinkling in Human Skin: A Randomized, Double-Blind, Placebo-Controlled Study. Nutrients.

[45] Gidley M.J., Yakubov G.E. (2019). Functional categorisation of dietary fibre in foods: Beyond ‘soluble’ vs. ‘insoluble’. Trends Food Sci. Technol..

[46] McRorie J.W., McKeown N.M. (2017). Understanding the Physics of Functional Fibers in the Gastrointestinal Tract: An Evidence-Based Approach to Resolving Enduring Misconceptions about Insoluble and Soluble Fiber. J. Acad. Nutr. Diet..

[47] Turner N.D., Lupton J.R. (2011). Dietary Fiber. Adv. Nutr..

[48] Soliman G.A. (2019). Dietary Fiber, Atherosclerosis, and Cardiovascular Disease. Nutrients.

[49] Hou J.K., Abraham B., El-Serag H. (2011). Dietary intake and risk of developing inflammatory bowel disease: A systematic review of the literature. Am. J. Gastroenterol..

[50] Lee Y.-H., Bae S.-C., Song G.-G. (2012). Omega-3 Polyunsaturated Fatty Acids and the Treatment of Rheumatoid Arthritis: A Meta-analysis. Arch. Med. Res..

[51] Kumar G.P., Khanum F. (2012). Neuroprotective potential of phytochemicals. Pharmacogn. Rev..

[52] Lust J. (2014). The Herb Book: The Most Complete Catalog of Herbs Ever Published.

[53] Embuscado M.E. (2015). Spices and herbs: Natural sources of antioxidants—A mini review. J. Funct. Foods.

[54] Borkar N., Saurabh S., Rathore K., Pandit A., Khandelwal K. (2015). An Insight on Nutraceuticals. PharmaTutor.

[55] Kechagia M., Basoulis D., Konstantopoulou S., Dimitriadi D., Gyftopoulou K., Skarmoutsou N., Fakiri E.M. (2013). Health Benefits of Probiotics: A Review. ISRN Nutr..

[56] Fuller R. (2012). Probiotics: The Scientific Basis.

[57] Al-Sheraji S.H., Ismail A., Manap M.Y., Mustafa S., Yusof R.M., Hassan F.A. (2013). Prebiotics as Functional Foods: A review. J. Funct. Foods.

[58] Caetano B.F.R., De Moura N.A., Almeida A.P.S., Dias M.C., Sivieri K., Barbisan L.F. (2016). Yacon (*Smallanthus sonchifolius*) as a Food Supplement: Health-Promoting Benefits of Fructooligosaccharides. Nutrients.

[59] Bailey R.L., Gahche J.J., Miller P.E., Thomas P.R., Dwyer J.T. (2013). Why US adults use dietary supplements. JAMA Intern. Med..

[60] Gupta S., Chauhan D., Mehla K., Sood P., Nair A. (2010). An Overview of Nutraceuticals: Current Scenario. J. Basic Clin. Pharm..

[61] Webb G.P. (2011). Dietary Supplements and Functional Foods.

[62] Nabarro L., Morris-Jones S., Moore D.A.J., Nabarro L., Morris-Jones S., Moore D.A.J. (2020). 7—Nutrition. Peter’s Atlas of Tropical Medicine and Parasitology.

[63] Anjali Garg V., Dhiman A., Dutt R., Ranga S. (2018). Health benefits of nutraceuticals. Pharm. Innov. J..

[64] Roadjanakamolson M., Suntornsuk W. (2010). Production of beta-carotene-enriched rice bran using solid-state fermentation of Rhodotorula glutinis. J. Microbiol. Biotechnol..

[65] Shekhar V., Jha A.K., Dangi J.S. Nutraceuticals: A Re-emerging Health Aid. Proceedings of the International Conference on Food, Biological and Medical Sciences (FBMS-2014).

[66] Kalra E.K. (2003). Nutraceutical-definition and introduction. AAPS Pharmsci..

[67] Swaroopa G., Srinath D. (2017). Nutraceuticals and their Health Benefits. Int. J. Pure Appl. Biosci..

[68] Sui X., Zhang Y., Zhou W. (2016). Bread fortified with anthocyanin-rich extract from black rice as nutraceutical sources: Its quality attributes and in vitro digestibility. Food Chem..

[69] Drake P.M.W., Szeto T.H., Paul M.J., Teh A.Y.-H., Ma J.K. (2017). Recombinant biologic products versus nutraceuticals from plants—A regulatory choice?. Br. J. Clin. Pharmacol..

[70] Alamgir A.N.M. (2017). Therapeutic Use of Medicinal Plants and Their Extracts. Pharmacognosy.

[71] Skinner M.A., Loh J.M.S., Hunter D.C., Zhang J. (2011). Gold kiwifruit (*Actinidia chinensis* ‘Hort16A’) for immune support. Proc. Nutr. Soc..

[72] Stonehouse W., Gammon C.S., Beck K.L., Conlon C.A., von Hurst P.R., Kruger R. (2012). Kiwifruit: Our daily prescription for health. Can. J. Physiol. Pharmacol..

[73] Shiby V.K., Mishra H.N. (2013). Fermented Milks and Milk Products as Functional Foods—A Review. Crit. Rev. Food Sci. Nutr..

[74] Vo T.-S., Kim S.-K. (2013). Fucoidans as a natural bioactive ingredient for functional foods. J. Funct. Foods.

[75] Latté K.P., Appel K.-E., Lampen A. (2011). Health benefits and possible risks of broccoli—An overview. Food Chem. Toxicol..

[76] Irw Jaswir I., Noviendri D., Hasrini R.F., Octavianti F. (2011). Carotenoids: Sources, medicinal properties and their application in food and nutraceutical industry. JMPR.

[77] Chauhan B., Kumar G., Kalam N., Ansari S.H. (2013). Current concepts and prospects of herbal nutraceutical: A review. J. Adv. Pharm. Technol. Res..

[78] Kispotta A., Srivastava M.K., Dutta M. (2012). Free radical scavenging activity of ethanolic extracts and determination of aloin from *Aloe vera* L. leaf extract. Int. J. Med. Aromat. Plants.

[79] Souyoul S.A., Saussy K.P., Lupo M.P. (2018). Nutraceuticals: A Review. Dermatol. Ther..

[80] Bailey R.L., Dodd K.W., Gahche J.J., Dwyer J.T., McDowell M.A., Yetley E.A., Sempos C.A., Burt V.L., Radimer K.L., Picciano M.F. (2010). Total folate and folic acid intake from foods and dietary supplements in the United States: 2003–2006. Am. J. Clin. Nutr..

[81] Areco V., Rivoira M.A., Rodriguez V., Marchionatti A.M., Carpentieri A., de Talamoni N.T. (2015). Dietary and pharmacological compounds altering intestinal calcium absorption in humans and animals. Nutr. Res. Rev..

[82] Martini M., Altomonte I., Licitra R., Salari F. (2018). Nutritional and Nutraceutical Quality of Donkey Milk. J. Equine Vet. Sci..

[83] Hossein-nezhad A., Holick M.F. (2013). Vitamin D for Health: A Global Perspective. Mayo Clin. Proc..

[84] Keservani R.K., Kesharwani R.K., Vyas N., Jain S., Sharma A.K. (2010). Nutraceutical and Functional Food as Future Food: A Review. Der Pharm. Lett..

[85] Prasanna P.H.P., Grandison A.S., Charalampopoulos D. (2014). Bifidobacteria in milk products: An overview of physiological and biochemical properties, exopolysaccharide production, selection criteria of milk products and health benefits. Food Res. Int..

[86] Shoaib M., Shehzad A., Omar M., Rakha A., Raza H., Sharif H.R., Shakeel A., Ansari A., Niazi S. (2016). Inulin: Properties, health benefits and food applications. Carbohydr. Polym..

[87] Golla U. (2018). Emergence of nutraceuticals as the alternative medications for pharmaceuticals. IJCAM.

[88] Dav G., Santilli F., Patrono C. (2010). Nutraceuticals in Diabetes and Metabolic Syndrome. Cardiovasc. Ther..

[89] Johnston T.P., Korolenko T.A., Pirro M., Sahebkar A. (2017). Preventing cardiovascular heart disease: Promising nutraceutical and non-nutraceutical treatments for cholesterol management. Pharmacol. Res..

[90] Kramer K., Hoppe P.-P., Packer L. (2001). Nutraceuticals in Health and Disease Prevention.

[91] Bele A.A., Khale A. (2013). An approach to a Nutraceutical. Asian J. Res. Chem..

[92] Gupta S.V., Pathak Y.V. (2019). Advances in Nutraceutical Applications in Cancer: Recent Research Trends and Clinical Applications.

[93] Sarkar F.H., Li Y., Wang Z., Kong D. (2010). The role of nutraceuticals in the regulation of Wnt and Hedgehog signaling in cancer. Cancer Metastasis Rev..

[94] De Mejia E.G., Dia V.P. (2010). The role of nutraceutical proteins and peptides in apoptosis, angiogenesis, and metastasis of cancer cells. Cancer Metastasis Rev..

[95] Shukla Y., George J. (2011). Combinatorial strategies employing nutraceuticals for cancer development. Ann. N. Y. Acad. Sci..

[96] Fathi N., Ahmadian E., Shahi S., Roshangar L., Khan H., Kouhsoltani M., Dizaj S.M., Sharifi S. (2019). Role of vitamin D and vitamin D receptor (VDR) in oral cancer. Biomed. Pharmacother..

[97] Vuolo L., Faggiano A., Colao A. (2012). Vitamin D and Cancer. Front. Endocrinol..

[98] Hii L.-W., Swee-Hua E.L., Leong C.-O., Swee-Yee C., Tan N.-P., Kok-Song L., Mai C.-W. (2019). The synergism of Clinacanthus nutans Lindau extracts with gemcitabine: Downregulation of anti-apoptotic markers in squamous pancreatic ductal adenocarcinoma. BMC Complement. Altern. Med..

[99] Bishehsari F., Engen P.A., Preite N.Z., Tuncil Y.E., Naqib A., Shaikh M., Rossi M., Wilber S., Green S.J., Hamaker B.R. (2018). Dietary Fiber Treatment Corrects the Composition of Gut Microbiota, Promotes SCFA Production, and Suppresses Colon Carcinogenesis. Genes.

[100] Gamallat Y., Meyiah A., Kuugbee E.D., Hago A.M., Chiwala G., Awadasseid A., Bamba D., Zhang X., Shang X., Luo F. (2016). Lactobacillus rhamnosus induced epithelial cell apoptosis, ameliorates inflammation and prevents colon cancer development in an animal model. Biomed. Pharmacother..

[101] Kuugbee E.D., Shang X., Gamallat Y., Bamba D., Awadasseid A., Suliman M.A., Zang S., Ma Y., Chiwala G., Xin Y. (2016). Structural Change in Microbiota by a Probiotic Cocktail Enhances the Gut Barrier and Reduces Cancer via TLR2 Signaling in a Rat Model of Colon Cancer. Dig. Dis. Sci..

[102] Raman M., Ambalam P., Kondepudi K.K., Pithva S., Kothari C., Patel A.T., Purama R.K., Dave J.M., Vyas B.R.M. (2013). Potential of probiotics, prebiotics and synbiotics for management of colorectal cancer. Gut Microbes..

[103] Juneja L.R., Kapoor M.P., Okubo T., Rao T. (2013). Green Tea Polyphenols: Nutraceuticals of Modern Life.

[104] Lee P.M.Y., Ng C.F., Liu Z.M., Ho W.M., Lee M.K., Wang F., Kan H.D., He Y.H., Ng S.S.M., Wong S.Y.S. (2017). Reduced prostate cancer risk with green tea and epigallocatechin 3-gallate intake among Hong Kong Chinese men. Prostate Cancer Prostatic Dis..

[105] Posadino A.M., Phu H.T., Cossu A., Giordo R., Fois M., Thuan D.T.B., Piga A., Sotgia S., Zinellu A., Carru C. (2017). Oxidative stress-induced Akt downregulation mediates green tea toxicity towards prostate cancer cells. Toxicol. Vitr..

[106] Huang J., Li F., Chen S., Shi Y., Wang X., Wang C., Meng Q.-h., Jiang C., Zhu Z.-y., Li C.-H. (2017). Green tea polyphenol induces significant cell death in human lung cancer cells. Trop. J. Pharm. Res..

[107] Leong H., Mathur P.S., Greene G.L. (2008). Inhibition of mammary tumorigenesis in the C3(1)/SV40 mouse model by green tea. Breast Cancer Res. Treat..

[108] Jian W., Fang S., Chen T., Fang J., Mo Y., Li D., Xiong S., Liu W., Song L., Shen J. (2019). A novel role of HuR in -Epigallocatechin-3-gallate (EGCG) induces tumour cells apoptosis. J. Cell Mol. Med..

[109] Kunnumakkara A.B., Anand P., Aggarwal B.B. (2008). Curcumin inhibits proliferation, invasion, angiogenesis and metastasis of different cancers through interaction with multiple cell signaling proteins. Cancer Lett..

[110] Vishvakarma N.K., Kumar A., Singh S.M. (2011). Role of curcumin-dependent modulation of tumor microenvironment of a murine T cell lymphoma in altered regulation of tumor cell survival. Toxicol. Appl. Pharmacol..

[111] Akkoç Y., Berrak Ö., Arısan E.D., Obakan P., Çoker-Gürkan A., Palavan-Ünsal N. (2015). Inhibition of PI3K signaling triggered apoptotic potential of curcumin which is hindered by Bcl-2 through activation of autophagy in MCF-7 cells. Biomed. Pharmacother..

[112] Li Y., Sun W., Han N., Zou Y., Yin D. (2018). Curcumin inhibits proliferation, migration, invasion and promotes apoptosis of retinoblastoma cell lines through modulation of miR-99a and JAK/STAT pathway. BMC Cancer.

[113] Gupta S.C., Kannappan R., Reuter S., Kim J.H., Aggarwal B.B. (2011). Chemosensitization of tumors by resveratrol. Ann. N. Y. Acad. Sci..

[114] Jiang Z., Chen K., Cheng L., Yan B., Qian W., Cao J., Li J., Wu E., Ma Q., Yang W. (2017). Resveratrol and cancer treatment: Updates. Ann. N. Y. Acad. Sci..

[115] Wu F., Cui L. (2017). Resveratrol suppresses melanoma by inhibiting NF-κB/miR-221 and inducing TFG expression. Arch. Dermatol. Res..

[116] Li W., Ma X., Li N., Liu H., Dong Q., Zhang J., Yang C., Liu Y., Liang Q., Zhang S. (2016). Resveratrol inhibits Hexokinases II mediated glycolysis in non-small cell lung cancer via targeting Akt signaling pathway. Exp. Cell Res..

[117] Karlsson S., Olausson J., Lundh D., Sögård P., Mandal A., Holmström K.-O., Stahel A., Bengtsson J., Larsson D. (2010). Vitamin D and prostate cancer: The role of membrane initiated signaling pathways in prostate cancer progression. J. Steroid Biochem. Mol. Biol..

[118] Lassed S., Deus C.M., Djebbari R., Zama D., Oliveira P.J., Rizvanov A.A., Dahdouh A., Benayache F., Benayache S. (2017). Protective Effect of Green Tea (*Camellia sinensis* (L.) Kuntze) against Prostate Cancer: From In Vitro Data to Algerian Patients. Evid. Based Complement Altern. Med..

[119] Yang K., Gao Z.-Y., Li T.-Q., Song W., Xiao W., Zheng J., Chen H., Chen G.-H., Zou H.-Y. (2019). Anti-tumor activity and the mechanism of a green tea (*Camellia sinensis*) polysaccharide on prostate cancer. Int. J. Biol. Macromol..

[120] Johnson J.J., Mukhtar H. (2007). Curcumin for chemoprevention of colon cancer. Cancer Lett..

[121] Umesalma S., Sudhandiran G. (2010). Differential Inhibitory Effects of the Polyphenol Ellagic Acid on Inflammatory Mediators NF-κB, iNOS, COX-2, TNF-α, and IL-6 in 1,2-Dimethylhydrazine-Induced Rat Colon Carcinogenesis. Basic Clin. Pharmacol. Toxicol..

[122] Akrout A., Gonzalez L.A., El Jani H., Madrid P.C. (2011). Antioxidant and antitumor activities of Artemisia campestris and Thymelaea hirsuta from southern Tunisia. Food Chem. Toxicol..

[123] Jayaprakasha G.K., Murthy K.N.C., Uckoo R.M., Patil B.S. (2013). Chemical composition of volatile oil from Citrus limettioides and their inhibition of colon cancer cell proliferation. Ind. Crops Prod..

[124] Mondal A., Gandhi A., Fimognari C., Atanasov A.G., Bishayee A. (2019). Alkaloids for cancer prevention and therapy: Current progress and future perspectives. Eur. J. Pharmacol..

[125] Shoeb M., MacManus S.M., Jaspars M., Trevidu J., Nahar L., Kong-Thoo-Lin P., Sarkere S.D. (2006). Montamine, a unique dimeric indole alkaloid, from the seeds of *Centaurea montana* (Asteraceae), and its in vitro cytotoxic activity against the CaCo2 colon cancer cells. Tetrahedron.

[126] Zhou J., Feng J.-H., Fang L. (2017). A novel monoterpenoid indole alkaloid with anticancer activity from Melodinus khasianus. Bioorg. Med. Chem. Lett..

[127] Chang H.-F., Yang L.-L. (2012). Gamma-Mangostin, a Micronutrient of Mangosteen Fruit, Induces Apoptosis in Human Colon Cancer Cells. Molecules.

[128] Prinz-langenohl R., Fohr I., Pietrzik K. (2001). Beneficial role for folate in the prevention of colorectal and breast cancer. Eur. J. Nutr..

[129] Modem S., DiCarlo S.E., Reddy T.R. (2012). Fresh Garlic Extract Induces Growth Arrest and Morphological Differentiation of MCF7 Breast Cancer Cells. Genes Cancer.

[130] Talib W.H. (2017). Consumption of garlic and lemon aqueous extracts combination reduces tumor burden by angiogenesis inhibition, apoptosis induction, and immune system modulation. Nutrition.

[131] Vijayakumar S., Malaikozhundan B., Saravanakumar K., Durán-Lara E.F., Wang M.-H., Vaseeharan B. (2019). Garlic clove extract assisted silver nanoparticle—Antibacterial, antibiofilm, antihelminthic, anti-inflammatory, anticancer and ecotoxicity assessment. J. Photochem. Photobiol. B.

[132] Kronski E., Fiori M.E., Barbieri O., Astigiano S., Mirisola V., Killian P.H., Bruno A., Pagani A., Rovera F., Pfeffer U. (2014). miR181b is induced by the chemopreventive polyphenol curcumin and inhibits breast cancer metastasis via down-regulation of the inflammatory cytokines CXCL1 and -2. Mol. Oncol..

[133] Simboli-campbell M., Narvaez C.J., Vanweelden K., Tenniswood M., Welsh J. (1997). Comparative effects of 1,25(OH)_2_D_3_ and EB1089 on cell cycle kinetics and apoptosis in MCF-7 breast cancer cells. Breast Cancer Res. Treat..

[134] Wang Q., Lee D., Sysounthone V., Chandraratna R.A.S., Christakos S., Korah R., Wieder R. (2001). 1,25-dihydroxyvitamin D_3_ and retonic acid analogues induce differentiation in breast cancer cells with function- and cell-specific additive effects. Breast Cancer Res. Treat..

[135] Zhu X., Xiong L., Zhang X., Shi N., Zhang Y., Ke J., Sun Z., Chen T. (2015). Lyophilized strawberries prevent 7,12-dimethylbenz[α]anthracene (DMBA)-induced oral squamous cell carcinogenesis in hamsters. J. Funct. Foods.

[136] Petiwala S.M., Johnson J.J. (2015). Diterpenes from rosemary (*Rosmarinus officinalis*): Defining their potential for anti-cancer activity. Cancer Lett..

[137] Vinothkumar V., Manoharan S., Sindhu G., Nirmal M.R., Vetrichelvi V. (2012). Geraniol modulates cell proliferation, apoptosis, inflammation, and angiogenesis during 7,12-dimethylbenz[a]anthracene-induced hamster buccal pouch carcinogenesis. Mol. Cell Biochem..

[138] Nair H.B., Sung B., Yadav V.R., Kannappan R., Chaturvedi M.M., Aggarwal B.B. (2010). Delivery of antiinflammatory nutraceuticals by nanoparticles for the prevention and treatment of cancer. Biochem. Pharmacol..

[139] Al-Okbi S.Y. (2014). Nutraceuticals of anti-inflammatory activity as complementary therapy for rheumatoid arthritis. Toxicol. Ind. Health.

[140] Vecchione R., Quagliariello V., Calabria D., Calcagno V., De Luca E., Iaffaioli R.V., Netti P.A. (2016). Curcumin bioavailability from oil in water nano-emulsions: In vitro and in vivo study on the dimensional, compositional and interactional dependence. J. Control. Release..

[141] Kunnumakkara A.B., Bordoloi D., Padmavathi G., Monisha J., Roy N.K., Prasad S., Aggarwal B.B. (2017). Curcumin, the golden nutraceutical: Multitargeting for multiple chronic diseases. Br. J. Pharmacol..

[142] Panahi Y., Sahebkar A., Amiri M., Davoudi S.M., Beiraghdar F., Hoseininejad S.L., Kolivand M. (2012). Improvement of sulphur mustard-induced chronic pruritus, quality of life and antioxidant status by curcumin: Results of a randomised, double-blind, placebo-controlled trial. Br. J. Nutr..

[143] Mijan M.A., Lim B.O. (2018). Diets, functional foods, and nutraceuticals as alternative therapies for inflammatory bowel disease: Present status and future trends. World J. Gastroenterol..

[144] Rodríguez-Cruz M., del Cruz-Guzmán O.R., Almeida-Becerril T., Solís-Serna A.D., Atilano-Miguel S., Sánchez-González J.R., Barbosa-Cortés L., Ruíz-Cruz E.D., Huicochea J.C., Cárdenas-Conejo A. (2018). Potential therapeutic impact of omega-3 long chain-polyunsaturated fatty acids on inflammation markers in Duchenne muscular dystrophy: A double-blind, controlled randomized trial. Clin. Nutr..

[145] Bansal P., Gupta S.K., Ojha S.K., Nandave M., Mittal R., Kumari S., Arya D.S. (2006). Cardioprotective effect of lycopene in the experimental model of myocardial ischemia-reperfusion injury. Mol. Cell. Biochem..

[146] Ojha S., Goyal S., Sharma C., Arora S., Kumari S., Arya D.S. (2013). Cardioprotective effect of lycopene against isoproterenol-induced myocardial infarction in rats. Hum. Exp. Toxicol..

[147] Song B., Liu K., Gao Y., Zhao L., Fang H., Li Y., Pei L., Xu Y. (2017). Lycopene and risk of cardiovascular diseases: A meta-analysis of observational studies. Mol. Nutr. Food Res..

[148] Srutkova D., Schwarzer M., Hudcovic T., Zakostelska Z., Drab V., Spanova A., Rittich B., Kozakova H., Schabussova I. (2015). Bifidobacterium longum CCM 7952 Promotes Epithelial Barrier Function and Prevents Acute DSS-Induced Colitis in Strictly Strain-Specific Manner. PLoS ONE.

[149] Sichetti M., De Marco S., Pagiotti R., Traina G., Pietrella D. (2018). Anti-inflammatory effect of multistrain probiotic formulation (*L. rhamnosus*, *B. lactis*, and *B. longum*). Nutrition.

[150] Liu X., Wu Y., Li F., Zhang D. (2015). Dietary fiber intake reduces risk of inflammatory bowel disease: Result from a meta-analysis. Nutr. Res..

[151] Lee K.-H., Park M., Ji K.-Y., Lee H.-Y., Jang J.-H., Yoon I.-J., Oh S.-S., Kim S.-M., Jeong Y.-H., Yun C.-H. (2014). Bacterial β-(1,3)-glucan prevents DSS-induced IBD by restoring the reduced population of regulatory T cells. Immunobiology.

[152] Cakir U., Tayman C., Serkant U., Yakut H.I., Cakir E., Ates U., Koyuncu I., Karaogul E. (2018). Ginger (*Zingiber officinale* Roscoe) for the treatment and prevention of necrotizing enterocolitis. J. Ethnopharmacol..

[153] El-Abhar H.S., Hammad L.N.A., Gawad H.S.A. (2008). Modulating effect of ginger extract on rats with ulcerative colitis. J. Ethnopharmacol..

[154] Lv J., Huang H., Yu L., Whent M., Niu Y., Shi H., Wang T.T.Y., Luthria D., Charles D., Yu L.L. (2012). Phenolic composition and nutraceutical properties of organic and conventional cinnamon and peppermint. Food Chem..

[155] Hassanzadeh P., Arbabi E., Atyabi F., Dinarvand R. (2016). The endocannabinoid system and NGF are involved in the mechanism of action of resveratrol: A multi-target nutraceutical with therapeutic potential in neuropsychiatric disorders. Psychopharmacology.

[156] Cui L., Feng L., Zhang Z.H., Jia X.B. (2014). The anti-inflammation effect of baicalin on experimental colitis through inhibiting TLR4/NF-κB pathway activation. Int. Immunopharmacol..

[157] Bitto A., Minutoli L., David A., Irrera N., Rinaldi M., Venuti F.S., Squadrito F., Altavilla D. (2012). Flavocoxid, a dual inhibitor of COX-2 and 5-LOX of natural origin, attenuates the inflammatory response and protects mice from sepsis. Crit. Care.

[158] Liu L., Shang Y., Li M., Han X., Wang J., Wang J. (2015). Curcumin ameliorates asthmatic airway inflammation by activating nuclear factor-E2-related factor 2/haem oxygenase (HO)-1 signalling pathway. Clin. Exp. Pharmacol. Physiol..

[159] Algandaby M.M., El-halawany A.M., Abdallah H.M., Alahdal A.M., Nagy A.A., Ashour O.M., Abdel-Naim A.B. (2016). Gingerol protects against experimental liver fibrosis in rats via suppression of pro-inflammatory and profibrogenic mediators. Naunyn Schmiedeberg Arch. Pharmacol..

[160] Zhang T., Su J., Guo B., Wang K., Li X., Liang G. (2015). Apigenin protects blood–brain barrier and ameliorates early brain injury by inhibiting TLR4-mediated inflammatory pathway in subarachnoid hemorrhage rats. Int. Immunopharmacol..

[161] Zhai W., Zhang Z., Xu N., Guo Y., Qiu C., Li C., Deng G.Z., Guo M.Y. (2016). Piperine Plays an Anti-Inflammatory Role in Staphylococcus aureus Endometritis by Inhibiting Activation of NF-κB and MAPK Pathways in Mice. Evid. Based Complement Altern. Med..

[162] Sahu B.D., Tatireddy S., Koneru M., Borkar R.M., Kumar J.M., Kuncha M., Srinivas R., Sunder R S., Sistla R. (2014). Naringin ameliorates gentamicin-induced nephrotoxicity and associated mitochondrial dysfunction, apoptosis and inflammation in rats: Possible mechanism of nephroprotection. Toxicol. Appl. Pharmacol..

[163] Kennedy-Feitosa E., Okuro R.T., Pinho Ribeiro V., Lanzetti M., Barroso M.V., Zin W.A., Porto L.C., Brito-Gitirana L., Valenca S.S. (2016). Eucalyptol attenuates cigarette smoke-induced acute lung inflammation and oxidative stress in the mouse. Pulm. Pharmacol. Ther..

[164] Fonsêca D., Salgado P., de Aragão Neto H., Golzio A., Caldas Filho M., Melo C., Leite F., Piuvezam M., Pordeus L., Barbosa Filho J. (2016). Ortho-eugenol exhibits anti-nociceptive and anti-inflammatory activities. Int. Immunopharmacol..

[165] Simioni C., Zauli G., Martelli A.M., Vitale M., Sacchetti G., Gonelli A., Neri L.M. (2018). Oxidative stress: Role of physical exercise and antioxidant nutraceuticals in adulthood and aging. Oncotarget.

[166] Wu Q., Liu L., Miron A., Klímová B., Wan D., Kuca K. (2016). The antioxidant, immunomodulatory, and anti-inflammatory activities of Spirulina: An overview. Arch. Toxicol. Arch. Toxikol..

[167] Trachootham D., Lu W., Ogasawara M.A., Valle N.R.-D., Huang P. (2008). Redox Regulation of Cell Survival. Antioxid. Redox Signal..

[168] Lushchak V.I. (2014). Free radicals, reactive oxygen species, oxidative stress and its classification. Chem. Biol. Interact..

[169] McCubrey J.A., Lertpiriyapong K., Steelman L.S., Abrams S.L., Yang L.V., Murata R.M., Rosalen P.L., Scalisi A., Neri L.M., Cocco L. (2017). Effects of resveratrol, curcumin, berberine and other nutraceuticals on aging, cancer development, cancer stem cells and microRNAs. Aging.

[170] Liguori I., Russo G., Curcio F., Bulli G., Aran L., Della-Morte D., Gargiulo G., Testa G., Cacciatore F., Bonaduce D. (2018). Oxidative stress, aging, and diseases. Clin. Interv. Aging..

[171] Naveen J., Baskaran V. (2018). Antidiabetic plant-derived nutraceuticals: A critical review. Eur. J. Nutr..

[172] Neha K., Haider M.R., Pathak A., Yar M.S. (2019). Medicinal prospects of antioxidants: A review. Eur. J. Med. Chem..

[173] Srinivasan K. (2014). Antioxidant Potential of Spices and Their Active Constituents. Crit. Rev. Food Sci. Nutr..

[174] Danwilai K., Konmun J., Sripanidkulchai B., Subongkot S. (2017). Antioxidant activity of ginger extract as a daily supplement in cancer patients receiving adjuvant chemotherapy: A pilot study. Cancer Manag. Res..

[175] Cao C., Pathak S., Patil K. (2018). Antioxidant Nutraceuticals: Preventive and Healthcare Applications.

[176] Tesoriere L., Allegra M., Butera D., Livrea M.A. (2004). Absorption, excretion, and distribution of dietary antioxidant betalains in LDLs: Potential health effects of betalains in humans. Am. J. Clin. Nutr..

[177] Krajka-Kuźniak V., Szaefer H., Ignatowicz E., Adamska T., Baer-Dubowska W. (2012). Beetroot juice protects against N-nitrosodiethylamine-induced liver injury in rats. Food Chem. Toxicol..

[178] Coles L.T., Clifton P.M. (2012). Effect of beetroot juice on lowering blood pressure in free-living, disease-free adults: A randomized, placebo-controlled trial. Nutr. J..

[179] Jeszka-Skowron M., Zgoła-Grześkowiak A., Stanisz E., Waśkiewicz A. (2017). Potential health benefits and quality of dried fruits: Goji fruits, cranberries and raisins. Food Chem..

[180] Chang S.K., Alasalvar C., Shahidi F. (2016). Review of dried fruits: Phytochemicals, antioxidant efficacies, and health benefits. J. Funct. Foods.

[181] Hernández-Alonso P., Bulló M., Salas-Salvadó J. (2016). Pistachios for Health. Nutr. Today.

[182] Bulló M., Juanola-Falgarona M., Hernández-Alonso P., Salas-Salvadó J. (2015). Nutrition attributes and health effects of pistachio nuts. Br. J. Nutr..

[183] Bolling B.W., Chen C.-Y.O., McKay D.L., Blumberg J.B. (2011). Tree nut phytochemicals: Composition, antioxidant capacity, bioactivity, impact factors. A systematic review of almonds, Brazils, cashews, hazelnuts, macadamias, pecans, pine nuts, pistachios and walnuts. Nutr. Res. Rev..

[184] Houston M.C. (2007). Treatment of hypertension with nutraceuticals, vitamins, antioxidants and minerals. Expert Rev. Cardiovasc. Ther..

[185] Riccioni G., Gammone M.A., Currenti W., D’Orazio N. (2018). Effectiveness and Safety of Dietetic Supplementation of a New Nutraceutical on Lipid Profile and Serum Inflammation Biomarkers in Hypercholesterolemic Patients. Molecules.

[186] Lyseng-Williamson K.A. (2012). Ezetimibe/Simvastatin: A Guide to its Clinical Use in Hypercholesterolemia. Am. J. Cardiovasc. Drugs.

[187] Cicero A.F.G., Colletti A., Bajraktari G., Descamps O., Djuric D.M., Ezhov M., Fras Z., Katsiki N., Langlois M., Latkovskis G. (2017). Lipid lowering nutraceuticals in clinical practice: Position paper from an International Lipid Expert Panel. Arch. Med. Sci..

[188] Brown A.W., Hang J., Dussault P.H., Carr T.P. (2010). Phytosterol Ester Constituents Affect Micellar Cholesterol Solubility in Model Bile. Lipids.

[189] Trautwein E.A., Koppenol W.P., Jong A., de Hiemstra H., Vermeer M.A., Noakes M., Luscombe-Marsh N.D. (2018). Plant sterols lower LDL-cholesterol and triglycerides in dyslipidemic individuals with or at risk of developing type 2 diabetes; a randomized, double-blind, placebo-controlled study. Nutr. Diabetes.

[190] Amir Shaghaghi M., Harding S.V., Jones P.J.H. (2014). Water dispersible plant sterol formulation shows improved effect on lipid profile compared to plant sterol esters. J. Funct. Foods.

[191] Malina D.M.T., Fonseca F.A., Barbosa S.A., Kasmas S.H., Machado V.A., França C.N., Borges N.C., Moreno R.A., Izar M.C. (2015). Additive effects of plant sterols supplementation in addition to different lipid-lowering regimens. J. Clin. Lipidol..

[192] Becker D.J., Gordon R.Y., Halbert S.C., French B., Morris P.B., Rader D.J. (2009). Red yeast rice for dyslipidemia in statin-intolerant patients: A randomized trial. Ann. Intern. Med..

[193] Verhoeven V., Van der Auwera A., Van Gaal L., Remmen R., Apers S., Stalpaert M., Wens J., Hermans N. (2015). Can red yeast rice and olive extract improve lipid profile and cardiovascular risk in metabolic syndrome? A double blind, placebo controlled randomized trial. BMC Complement Altern. Med..

[194] Yang C.W., Mousa S.A. (2012). The effect of red yeast rice (*Monascus purpureus*) in dyslipidemia and other disorders. Complement Ther. Med..

[195] Biagi M., Minoretti P., Bertona M., Emanuele E. (2018). Effects of a nutraceutical combination of fermented red rice, liposomal berberine, and curcumin on lipid and inflammatory parameters in patients with mild-to-moderate hypercholesterolemia: An 8-week, open-label, single-arm pilot study. Arch. Med. Sci. Atheroscler. Dis..

[196] Xiong Z., Cao X., Wen Q., Chen Z., Cheng Z., Huang X., Zhang Y., Long C., Zhang Y., Huang Z. (2019). An overview of the bioactivity of monacolin K/lovastatin. Food Chem. Toxicol..

[197] Surampudi P., Enkhmaa B., Anuurad E., Berglund L. (2016). Lipid Lowering with Soluble Dietary Fiber. Curr. Atheroscler. Rep..

[198] Pirillo A., Catapano A.L. (2015). Berberine, a plant alkaloid with lipid- and glucose-lowering properties: From in vitro evidence to clinical studies. Atherosclerosis.

[199] Kim M., Kim Y. (2010). Hypocholesterolemic effects of curcumin via up-regulation of cholesterol 7a-hydroxylase in rats fed a high fat diet. Nutr. Res. Pract..

[200] Anadón A., Martínez-Larrañaga M.R., Ares I., Martínez M.A., Gupta R.C. (2016). Interactions between Nutraceuticals/Nutrients and Therapeutic Drugs. Nutraceuticals.

[201] Gul K., Singh A.K., Jabeen R. (2016). Nutraceuticals and Functional Foods: The Foods for the Future World. Crit. Rev. Food Sci. Nutr..

[202] Gil F., Hernández A.F., Martín-Domingo M.C., Gupta R.C. (2016). Toxic Contamination of Nutraceuticals and Food Ingredients. Nutraceuticals.

[203] Gupta R.C., Srivastava A., Lall R., Nicolotti O. (2018). Toxicity Potential of Nutraceuticals. Computational Toxicology: Methods and Protocols (Methods in Molecular Biology).

[204] Gupta R.C. (2016). Nutraceuticals: Efficacy, Safety and Toxicity.

[205] Girdhar S., Pandita D., Girdhar A., Lather V. (2017). Safety, Quality and Regulatory Aspects of Nutraceuticals. Appl. Clin. Res. Clin. Trials Regul. Aff..

[206] Ayaz M., Ullah F., Sadiq A., Ullah F., Ovais M., Ahmed J., Devkota H.P. (2019). Synergistic interactions of phytochemicals with antimicrobial agents: Potential strategy to counteract drug resistance. Chem. Biol. Interact..

[207] Guo X., Mei N. (2016). *Aloe vera*: A review of toxicity and adverse clinical effects. J. Environ. Sci. Health C.

[208] Boudreau M.D., Mellick P.W., Olson G.R., Felton R.P., Thorn B.T., Beland F.A. (2013). Toxicology and carcinogenesis studies of a nondecolorized [corrected] whole leaf extract of Aloe barbadensis Miller (*Aloe vera*) in F344/N rats and B6C3F1 mice (drinking water study). Natl. Toxicol. Program. Tech. Rep. Ser..

[209] Pandiri A.R., Sills R.C., Hoenerhoff M.J., Peddada S.D., Ton T.-V.T., Hong H.-H.L., Flake G.P., Malarkey D.E., Olson G.R., Pogribny I.P. (2011). *Aloe vera* Non-Decolorized Whole Leaf Extract-Induced Large Intestinal Tumors in F344 Rats Share Similar Molecular Pathways with Human Sporadic Colorectal Tumors. Toxicol. Pathol..

[210] Shao A., Broadmeadow A., Goddard G., Bejar E., Frankos V. (2013). Safety of purified decolorized (low anthraquinone) whole leaf *Aloe vera* (L) Burm. f. juice in a 3-month drinking water toxicity study in F344 rats. Food Chem. Toxicol..

[211] Williams L.D., Burdock G.A., Shin E., Kim S., Jo T.H., Jones K.N., Matulka R.A. (2010). Safety studies conducted on a proprietary high-purity *Aloe vera* inner leaf fillet preparation, Qmatrix^®^. Reg. Toxicol. Pharmacol..

[212] Sehgal I., Winters W.D., Scott M., Kousoulas K. (2013). An in vitro and in vivo toxicologic evaluation of a stabilized *Aloe vera* gel supplement drink in mice. Food Chem. Toxicol..

[213] Paes-Leme A.A., Motta E.S., De Mattos J.C.P., Dantas F.J.S., Bezerra R.J.A.C., Caldeira-de-Araujo A. (2005). Assessment of *Aloe vera* (L.) genotoxic potential on Escherichia coli and plasmid DNA. J. Ethnopharmacol..

[214] Chiang J.-H., Yang J.-S., Ma C.-Y., Yang M.-D., Huang H.-Y., Hsia T.-C., Kuo H.-M., Wu P.-P., Lee T.-H., Chung J.-G. (2011). Danthron, an Anthraquinone Derivative, Induces DNA Damage and Caspase Cascades-Mediated Apoptosis in SNU-1 Human Gastric Cancer Cells through Mitochondrial Permeability Transition Pores and Bax-Triggered Pathways. Chem. Res. Toxicol..

[215] National Toxicology Program (2013). Ntp Technical Report on the Toxicology and Carcinogenesis Studies of Ginkgo Biloba Extract (cas No. 90045-36-6) in F344/N Rats and B6c3f1/N Mice (Gavage Studies).

[216] Lin H., Guo X., Zhang S., Dial S.L., Guo L., Manjanatha M.G., Moore M.M., Mei N. (2014). Mechanistic Evaluation of Ginkgo biloba Leaf Extract-Induced Genotoxicity in L5178Y Cells. Toxicol. Sci..

[217] Maeda J., Kijima A., Inoue K., Ishii Y., Ichimura R., Takasu S., Kuroda K., Matsushita K., Kodama Y., Saito N. (2014). In Vivo Genotoxicity of Ginkgo Biloba Extract in gpt Delta Mice and Constitutive Androstane Receptor Knockout Mice. Toxicol. Sci..

[218] Resende F.A., Vilegas W., Dos Santos L.C., Varanda E.A. (2012). Mutagenicity of Flavonoids Assayed by Bacterial Reverse Mutation (Ames) Test. Molecules.

[219] National Toxicology Program (2010). Toxicology and Carcinogenesis Studies of Goldenseal Root Powder (Hydrastis canadensis) in F344/N Rats and B6c3f1 Mice (Feed Studies).

[220] Chen S., Wan L., Couch L., Lin H., Li Y., Dobrovolsky V.N., Mei N., Guo L. (2013). Mechanism study of goldenseal-associated DNA damage. Toxicol. Lett..

[221] Ajayi A.M., Umukoro S., Ben-Azu B., Adzu B., Ademowo O.G. (2017). Toxicity and Protective Effect of Phenolic-Enriched Ethylacetate Fraction of *Ocimum gratissimum* (Linn.) Leaf against Acute Inflammation and Oxidative Stress in Rats. Drug Dev. Res..

[222] Arslan Burnaz N., Küçük M., Akar Z. (2017). An on-line HPLC system for detection of antioxidant compounds in some plant extracts by comparing three different methods. J. Chromatogr. B.

[223] Gulati K., Anand R., Ray A., Gupta R.C. (2016). Nutraceuticals as Adaptogens: Their Role in Health and Disease. Nutraceuticals.

[224] Sobrinho A.P., Minho A.S., Ferreira L.L.C., Martins G.R., Boylan F., Fernandes P.D. (2017). Characterization of anti-inflammatory effect and possible mechanism of action of Tibouchina granulosa. J. Pharm. Phramacol..

[225] Wang K., Gupta R.C. (2016). Adverse Reaction Prediction and Pharmacovigilance of Nutraceuticals: Examples of Computational and Statistical Analysis on Big Data. Nutraceuticals.

[226] Gonçalves R.F.S., Martins J.T., Duarte C.M.M., Vicente A.A., Pinheiro A.C. (2018). Advances in nutraceutical delivery systems: From formulation design for bioavailability enhancement to efficacy and safety evaluation. Trends Food Sci. Technol..

[227] Peterson J.D., Gupta R.C. (2016). Noninvasive In Vivo Optical Imaging Models for Safety and Toxicity Testing. Nutraceuticals.

[228] Barnett R.E., Bailey D.C., Hatfield H.E., Fitsanakis V.A., Gupta R.C. (2016). Caenorhabditis elegans: A Model Organism for Nutraceutical Safety and Toxicity Evaluation. Nutraceuticals.

[229] Bian W.-P., Pei D.-S., Gupta R.C. (2016). Zebrafish Model for Safety and Toxicity Testing of Nutraceuticals. Nutraceuticals.

[230] Krishna G., Gopalakrishnan G., Gupta R.C. (2016). Alternative In Vitro Models for Safety and Toxicity Evaluation of Nutraceuticals. Nutraceuticals.

[231] Gonzalez-Suarez I., Martin F., Hoeng J., Peitsch M.C., Gupta R.C. (2016). Mechanistic Network Models in Safety and Toxicity Evaluation of Nutraceuticals. Nutraceuticals.

[232] Kadakkuzha B.M., Liu X., Swarnkar S., Chen Y., Gupta R.C. (2016). Genomic and Proteomic Mechanisms and Models in Toxicity and Safety Evaluation of Nutraceuticals. Nutraceuticals.

[233] Mouly S., Lloret-Linares C., Sellier P.-O., Sene D., Bergmann J.-F. (2017). Is the clinical relevance of drug-food and drug-herb interactions limited to grapefruit juice and Saint-John’s Wort?. Pharmacol. Res..

[234] Mooiman K.D., Maas-Bakker R.F., Hendrikx J.J.M.A., Bank P.C.D., Rosing H., Beijnen J.H., Schellens J.H.M., Meijerman I. (2014). The effect of complementary and alternative medicines on CYP3A4-mediated metabolism of three different substrates: 7-benzyloxy-4-trifluoromethyl-coumarin, midazolam and docetaxel. J. Pharm. Phramacol..

[235] Oh H.-A., Lee H., Kim D., Jung B.H. (2017). Development of GC-MS based cytochrome P450 assay for the investigation of multi-herb interaction. Anal. Biochem..

[236] Zhang L., Sparreboom A. (2017). Predicting transporter-mediated drug interactions: Commentary on: “Pharmacokinetic evaluation of a drug transporter cocktail consisting of digoxin, furosemide, metformin and rosuvastatin” and “Validation of a microdose probe drug cocktail for clinical drug interaction assessments for drug transporters and CYP3A”. Clin. Pharmacol. Ther..

[237] Gaudineau C., Beckerman R., Welbourn S., Auclair K. (2004). Inhibition of human P450 enzymes by multiple constituents of the Ginkgo biloba extract. Biochem. Biophys. Res. Commun..

[238] Shao F., Zhang H., Xie L., Chen J., Zhou S., Zhang J., Lv J., Hao W., Ma Y., Liu Y. (2017). Pharmacokinetics of ginkgolides A, B and K after single and multiple intravenous infusions and their interactions with midazolam in healthy Chinese male subjects. Eur. J. Clin. Pharmacol..

[239] Unger M., Frank A. (2004). Simultaneous determination of the inhibitory potency of herbal extracts on the activity of six major cytochrome P450 enzymes using liquid chromatography/mass spectrometry and automated online extraction. Rapid Commun. Mass Spetrom..

[240] Dürr D., Stieger B., Kullak-Ublick G.A., Rentsch K.M., Steinert H.C., Meier P.J., Fattinger K. (2000). St John’s Wort induces intestinal P-glycoprotein/MDR1 and intestinal and hepatic CYP3A4. Clin. Pharmacol. Ther..

[241] Goey A.K.L., Mooiman K.D., Beijnen J.H., Schellens J.H.M., Meijerman I. (2013). Relevance of in vitro and clinical data for predicting CYP3A4-mediated herb–drug interactions in cancer patients. Cancer Treat. Rev..

[242] Dormán G., Flachner B., Hajdú I., András C.D., Gupta R.C. (2016). Target Identification and Polypharmacology of Nutraceuticals. Nutraceuticals.

[243] Herr M., Grondin H., Sanchez S., Armaingaud D., Blochet C., Vial A., Denormandie P., Ankri J. (2017). Polypharmacy and potentially inappropriate medications: A cross-sectional analysis among 451 nursing homes in France. Eur. J. Clin. Pharmacol..

[244] Yang X., Li W., Sun Y., Guo X., Huang W., Peng Y., Zheng J. (2017). Comparative Study of Hepatotoxicity of Pyrrolizidine Alkaloids Retrorsine and Monocrotaline. Chem. Res. Toxicol..

[245] Merz K.-H., Schrenk D. (2016). Interim relative potency factors for the toxicological risk assessment of pyrrolizidine alkaloids in food and herbal medicines. Toxicol. Lett..

[246] Panter K.E., Welch K.D., Gardner D.R., Gupta R.C. (2019). Poisonous Plants: Biomarkers for Diagnosis. Nutraceuticals.

[247] Preliasco M., Gardner D., Moraes J., González A.C., Uriarte G., Rivero R. (2017). Senecio grisebachii Baker: Pyrrolizidine alkaloids and experimental poisoning in calves. Toxicon.

[248] Dlugaszewska J., Ratajczak M., Kamińska D., Gajecka M. (2019). Are dietary supplements containing plant-derived ingredients safe microbiologically?. Saudi Pharm. J..

[249] Bugno A., Almodovar A.A.B., Pereira T.C., Pinto T. (2006). de, J.A.; Sabino, M. Occurrence of toxigenic fungi in herbal drugs. Braz. J. Microbiol..

[250] Prado G., Altoé A.F., Gomes T.C.B., Leal A.S., Morais V.A.D., Oliveira M.S., Ferreira M.B., Gomes M.B., Paschoal F.N., Souza R.V.S. (2012). Occurrence of aflatoxin B1 in natural products. Braz. J. Microbiol..

[251] Sarma H., Deka S., Deka H., Saikia R.R., Whitacre D.M. (2011). Accumulation of Heavy Metals in Selected Medicinal Plants. Reviews of Environmental Contamination and Toxicology.

[252] Tong M., Gao W., Jiao W., Zhou J., Li Y., He L., Hou R. (2017). Uptake, Translocation, Metabolism, and Distribution of Glyphosate in Nontarget Tea Plant (*Camellia sinensis* L.). J. Agric. Food Chem..

[253] Hasler C.M. (2005). Regulation of Functional Foods and Nutraceuticals: A Global Perspective.

[254] Jacobo-Velázquez D.A., del Rosario Cuéllar-Villarreal M., Welti-Chanes J., Cisneros-Zevallos L., Ramos-Parra P.A., Hernández-Brenes C. (2017). Nonthermal processing technologies as elicitors to induce the biosynthesis and, accumulation of nutraceuticals in plant foods. Trends Food Sci. Technol..

[255] Del Cuéllar-Villarreal M.R., Ortega-Hernández E., Becerra-Moreno A., Welti-Chanes J., Cisneros-Zevallos L., Jacobo-Velázquez D.A. (2016). Effects of ultrasound treatment and storage time on the extractability and biosynthesis of nutraceuticals in carrot (*Daucus carota*). Postharvest Biol. Technol..

[256] Wu J., Lin L. (2002). Ultrasound-Induced Stress Responses of Panax ginsengCells: Enzymatic Browning and Phenolics Production. Biotechnol. Prog..

[257] Yu J., Engeseth N.J., Feng H. (2016). High Intensity Ultrasound as an Abiotic Elicitor—Effects on Antioxidant Capacity and Overall Quality of Romaine Lettuce. Food Bioprocess Technol..

[258] Ortega V.G., Ramírez J.A., Velázquez G., Tovar B., Mata M., Montalvo E. (2013). Effect of high hydrostatic pressure on antioxidant content of “Ataulfo” mango during postharvest maturation. Food Sci. Technol..

[259] Galindo F.G., Dejmek P., Lundgren K., Rasmusson A.G., Vicente A., Moritz T. (2009). Metabolomic evaluation of pulsed electric field-induced stress on potato tissue. Planta.

[260] Anita S., Mangesh T., Prasad V.S., SinghMeera C. (2013). Nutraceuticals-Global Status and Applications: A Review. https://www.semanticscholar.org/paper/Nutraceuticals-Global-status-and-applications-%3A-a-Anita-Mangesh/a1e4ce0f21e585b554e86b203f3bd8166b1cb112.

[261] Santini A., Tenore G.C., Novellino E. (2017). Nutraceuticals: A paradigm of proactive medicine. Eur. J. Pharm. Sci..

[262] Brower V. (2005). A nutraceutical a day may keep the doctor away. EMBO Rep..

[263] Mechanick J.I., Brett E.M. (2005). Nutrition and the chronically critically ill patient. Curr. Opin. Clin. Nutr. Metab. Care.

[264] Blades M. (2000). Functional foods or nutraceuticals. Nutr. Food Sci..

[265] Pandey M., Verma R.K., Saraf S.A. (2010). Nutraceuticals: New era of medicine and health. Asian J. Pharm. Clin. Res..

[266] Rudra S.G., Nishad J., Jakhar N., Kaur C. (2015). Food Industry Waste: Mine of Nutraceuticals. Int. J. Sci..

[267] Camacho F., Macedo A., Malcata F. (2019). Potential Industrial Applications and Commercialization of Microalgae in the Functional Food and Feed Industries: A Short Review. Mar. Drugs.

[268] Schröder M.J.A. (2003). Food Additives, Functional Food Ingredients and Food Contaminants. Food Quality and Consumer Value: Delivering Food That Satisfies.

